# Single-cell RNA sequencing reveals the evolution of the immune landscape during perihematomal edema progression after intracerebral hemorrhage

**DOI:** 10.1186/s12974-024-03113-8

**Published:** 2024-05-28

**Authors:** Peng Zhang, Cong Gao, Qiang Guo, Dongxu Yang, Guangning Zhang, Hao Lu, Liman Zhang, Guorong Zhang, Daojing Li

**Affiliations:** 1 Department of Clinical Medicine, Jining Medical University, Jining, China; 2 Department of Emergency Stroke, Affiliated Hospital of Jining Medical University, Jining, China; 3 Department of Neurosurgery, Affiliated Hospital of Jining Medical University, Jining, China; 4 Department of Pathology, Affiliated Hospital of Jining Medical University, Jining, China; 5 Department of Neurology, Affiliated Hospital of Jining Medical University, Jining, China

**Keywords:** Intracerebral hemorrhage, Single cell RNA sequencing, Perihematomal edema, Immune cell, Osteopontin

## Abstract

**Background:**

Perihematomal edema (PHE) after post-intracerebral hemorrhage (ICH) has complex pathophysiological mechanisms that are poorly understood. The complicated immune response in the post-ICH brain constitutes a crucial component of PHE pathophysiology. In this study, we aimed to characterize the transcriptional profiles of immune cell populations in human PHE tissue and explore the microscopic differences between different types of immune cells.

**Methods:**

9 patients with basal ganglia intracerebral hemorrhage (hematoma volume 50-100 ml) were enrolled in this study. A multi-stage profile was developed, comprising Group1 (*n* = 3, 0–6 h post-ICH, G1), Group2 (*n* = 3, 6–24 h post-ICH, G2), and Group3 (*n* = 3, 24–48 h post-ICH, G3). A minimal quantity of edematous tissue surrounding the hematoma was preserved during hematoma evacuation. Single cell RNA sequencing (scRNA-seq) was used to map immune cell populations within comprehensively resected PHE samples collected from patients at different stages after ICH.

**Results:**

We established, for the first time, a comprehensive landscape of diverse immune cell populations in human PHE tissue at a single-cell level. Our study identified 12 microglia subsets and 5 neutrophil subsets in human PHE tissue. What’s more, we discovered that the secreted phosphoprotein-1 (SPP1) pathway served as the basis for self-communication between microglia subclusters during the progression of PHE. Additionally, we traced the trajectory branches of different neutrophil subtypes. Finally, we also demonstrated that microglia-produced osteopontin (OPN) could regulate the immune environment in PHE tissue by interacting with CD44-positive cells.

**Conclusions:**

As a result of our research, we have gained valuable insight into the immune-microenvironment within PHE tissue, which could potentially be used to develop novel treatment modalities for ICH.

**Supplementary Information:**

The online version contains supplementary material available at 10.1186/s12974-024-03113-8.

## Introduction

Intracerebral hemorrhage (ICH) is one of the most critical and severe illnesses, posing a significant threat to global health due to its high rates of disability and mortality [1–3]. ICH initially causes injury through the physical disruption of the initial hemorrhage and hematoma expansion, and it also leads to secondary brain injury by the development of perihematomal edema (PHE) [4]. The formation of PHE occurs within 1–4 h after ICH, with subsequent progression for up to 2–3 weeks after that [5]. However, the specific pathophysiological mechanisms of PHE formation are complex and poorly understood. Previous studies have shown that PHE enhances the mass effect induced by initial hematoma and causes direct damage to brain tissue via blood-brain barrier (BBB) dysfunction and imbalanced osmotic gradients, leading to neurological deterioration [6]. Furthermore, PHE progression is closely related to adverse clinical outcomes and prognosis of patients with ICH [7]. Therefore, ICH remains inadequately controlled by current treatments. However, the emergence of immunomodulatory medications has reignited the hope for ICH treatment. Considering PHE’s role in secondary brain injury, developing effective immunomodulatory treatments targeting PHE might offer a new therapeutic modality for ICH.

Three randomized controlled trials have demonstrated that immunomodulatory drugs, such as Minocycline and Deferoxamine, aimed at targeting immune cells in ICH, have been unsuccessful in clinical settings [8–10]. The increasingly clear consensus is that we need to have a deeper understanding of the different roles of various immune subgroups in PHE, whether beneficial or harmful. Nevertheless, experimental research on immune cells related to stroke largely relies on previous knowledge, such as markers and phenotypes of immune cell populations defined in other research fields [11]. Conventional experimental techniques, such as immunofluorescent staining and flow cytometry, may present limitations in accurately identifying distinct immune cell subpopulations and conducting an impartial examination of the intricate immune cell phenotypes elicited by ICH. Therefore, a comprehensive understanding of the diversity within immune cells in PHE is crucial for developing effective and targeted immunotherapeutic modalities.

In this study, we aimed to analyze the immune cell composition in PHE tissue through single-cell RNA sequencing (scRNA-seq). Currently, scRNA-seq has been widely used to identify immune cell subsets specific to certain pathological conditions in the brain, including multiple sclerosis, epilepsy, as well as Alzheimer’s disease [12–14]. However, less is known about the heterogeneity of immune cells in ICH, especially in the setting of the human fresh brain. Given that the immune cell landscape at the single-cell level in PHE tissues during the progression of ICH still needs futher characterization. Therefore, this study aims to use scRNA-seq to de novo characterize the evolution process of immune cells in PHE tissues using transcriptional profiling of human immune cells.

## Materials and methods

### Patient inclusion and sample collection

The human brain tissue collection and research protocols were approved by the Affiliated Hospital of Jining Medical University and the Institutional Review Board (ethics committee, ethical code 2023-06-C004). Eligible subjects gave informed written consent. After quality control, a total of 9 samples were included in the analyses. The median age of the patients was 56.9 years, and the cohort consisted of 4 females and 5 males, whose detailed information was included in Table 1. Patient inclusion and exclusion criteria were as follows: (1) > 18 years old, (2) Intracerebral hemorrhage in the basal ganglia requires hematoma evacuation, (3) Hemorrhage volume: 50 to 100 ml, (4) With a history of hypertension. Exclusion criteria: (1) Patients who exhibiting pre-existing brain diseases, such as infections, traumatic cerebral hemorrhage, cerebrovascular malformations, aneurysms, cavernomas, (2) Pathologically confirmed amyloid angiopathy, (3) Patients undergoing immunosuppressive or immunomodulatory therapy, (4) Patients reported any cancer site. Finally, we recruited total 9 eligible participants and a multi-stage profile was developed, comprising Group1 (*n* = 3, 0–6 h post-ICH, G1), Group2 (*n* = 3, 6–24 h post-ICH, G2), and Group3 (*n* = 3, 24–48 h post-ICH, G3) [15, 16]. The surgical technique employed was the transsylvian-transinsular approach for hematoma removal. Fresh brain tissues of perihematomal regions were collected from patients undergoing hematoma evacuation after onset [17–19]. The brain tissue utilized in this study was obtained from the peristomal region adjacent to the hematoma cavity, with a mass of approximately 200 mg to conduct subsequent scRNA sequencing analyses.

**Table 1:** Baseline Values

Characteristics	G1 (n=3)	G2 (n=3)	G3 (n=3)
Age （mean±SD）	61±5.57	63±5.51	62±12.6
Male	33.33%	66.66%	66.66%
Hypertension	100%	100%	100%
Stroke	0	0	0
Diabetes	0	33.33%	0
GCS score	7.67±1.52	6.67±2.08	6.00±1.73
Systolic Blood Pressure	188±11.8	185±20.0	175±25.0
Hematoma Volume	53±4.36	55±4.58	55±4.04

### ScRNA sequencing

According to the manufacture’s introduction, Single-cell RNA-seq libraries were constructed using Single Cell 3’ Library and Gel Bead Kit V3.1. The libraries were finally sequenced using an Illumina Novaseq6000 sequencer with a sequencing depth of at least 100,000 reads per cell with pair-end 150 bp (PE150) strategy (performed by CapitalBio Technology, Beijing). After data preprocessing, the sequencing results were used to conduct Gene Set Variation Analysis (GSVA), Gene Set Enrichment Analysis (GSEA), single-cell regulatory network inference and clustering (SCENIC) Analysis, Monocle2 Pseudotime Analysis, Cell-Cell Communication Analysis. The details of post sequencing analyses are recorded in the Supplementary file “Materials and Methods”.

### Immunofluorescence staining

Immunohistochemistry was performed on 6-mm frozen sections. After the slides were brought to room temperature for 20 min, the tissue was fixed in 4% paraformaldehyde for 10 min and permeabilized in 0.3% Triton X for 5 min. After blocking with 5% bovine serum albumin for 30 min, the sections were incubated with primary antibodies overnight at 4 °C. The next day they were washed in PBS and then incubated for 60 min at room temperature with species-appropriate fluorochrome-conjugated secondary antibodies. The primary antibodies used were goat-anti Iba1 (1:200; Abcam ab5076); rabbit-anti OPN (1:250; Abcam ab63856); rat-anti CD44 (1:200, Abcam ab119348). After elution with PBS, they were incubated with donkey anti rabbit, donkey anti rat and donkey anti goat secondary antibodies. Finally, the anti fluorescence quenching agent containing DAPI is used for sealing.

## Results

### Single-cell transcriptome profiling reveals the heterogeneity of immune and non-immune cells after ICH

**Fig. 1:**
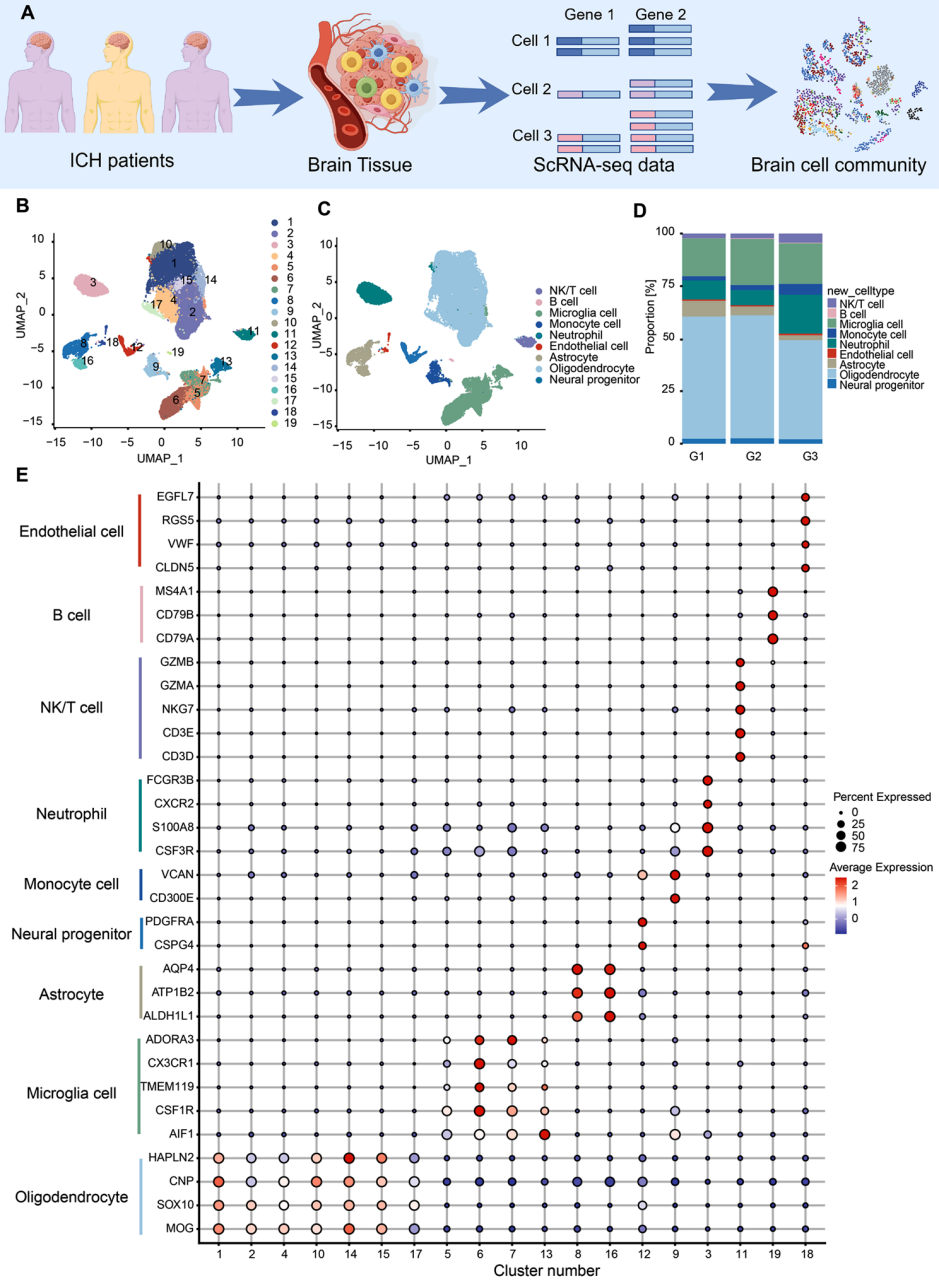
Single-cell profiling of diverse immune cells from three groups. **(A)** Overview of the study workflow. **(B)** Position of clusters on the UMAP map. Color represents the cluster ID. **(C)** Phenotype of clusters on the UMAP map. Different colors represent 9 clusters (cell types) respectively, including microglia, astrocytes, oligodendrocytes, neural progenitors, monocytes, neutrophils, NK/T cells, B cells, and endothelial cells. **(D)** Proportions of all cell types in each group during PHE progression. **(E)** Representative cell type marker genes (y-axis) with the percent of cells that express a gene (size of dot) in each cluster (distributed along the x-axis) and the average expression level (color intensity) are shown for microglia (*AIF1*, *CSF1R*, *TMEM119*, and *CX3CR1*), astrocytes (*AQP4*, *ATP1B2*, and *ALDH1L1*), oligodendrocyte (*MOG* and *SOX10*), neural progenitor (*PDGFRA*), monocyte (*VCAN* and *CD300E*), neutrophil (*CSF3R* and *S100A8*), NK/T cell (*GZMA*, *NKG7*, *CD3D*, and *CD3E*), B cell (*CD79B* and *MS4A1*) and endothelial cells (*VWF* and *CLDN5*) for each cluster

In order to reveal the changes in immune cells during the progression of ICH, we collected 10×Genomics scRNA-seq datasets from fresh PHE samples, which were resected from brain tissue in the vicinity of hematoma during the evacuation of the hematoma. We constructed a multi-stage profile including Group1 (*n* = 3, 0–6 h after ICH, G1), Group2 (*n* = 3, 6–24 h after ICH, G2), and Group3 (*n* = 3, 24–48 h after ICH, G3) (Fig. 1A). After quality control, a total of 3152–5823 cells were retained, with an average of 2639–4716 genes per cell and an average of 9535–24,244 unique molecular identifiers (UMIs) per cell for the subsequent analysis. Following that, we clustered and visualized various cell types refering to their relative gene expression levels using uniform manifold approximation and projection (UMAP), an unsupervised nonlinear dimensionality reduction algorithm. Graph-based Louvain clustering algorithms (with a resolution value of 0.4) were used to cluster all cells into subsets resulting in 19 clusters (Fig. 1B). Based on marker gene expression levels of microglia (*AIF1*, *CSF1R*, *TMEM119*, *CX3CR1*), clusters 5, 6, 7 and 13 were identified as microglial cells (Fig. 1C and E). Clusters 8 and 16 expressed genes (*ALDH1L1*, *ATP1B2* and *AQP4*) specific to astrocytes (Fig. 1C and E). Within the infiltrating immune cell subsets, cluster 3 had neutrophil marker genes (*CSF3R*, *S100A8*, *CXCR2* and *FCGR3B*) and cluster 9 had monocyte marker genes (*CD300E* and *VCAN*) (Fig. 1C and E). Cluster 11 expressed genes (*CD3D*, *CD3E*, *NKG7*, *GZMA* and *GZMB*) specific to cells of NK/T cells, while cluster 19 expressed genes (*CD79A*, *CD79B* and *MS4A1*) specific to B cells (Fig. 1C and E). Additionally, clusters 1, 2, 4, 10, 14, 15 and 17 expressed genes (*MOG*, *SOX10*, *CNP* and *HAPLN2*) specific to oligodendrocytes, and cluster 12 expressed genes (*CSPG4* and *PDGFRA*) specific to neural progenitor cells (Fig. 1C and E). Cluster 18 expressed endothelial cell marker genes (*CLDN5*, *VWF*, *RGS5* and *EGFL7*). Therefore, we identified this cluster as endothelial cells (Fig. 1C and E). Furthermore, neutrophils, NK/T cells, and monocyte cells were present at all time points and increased to reach their highest level at G3 (Fig. 1D), a timepoint that appears crucial to investigating ICH-induced immune cell changes [20]. Microglia and neutrophils, as the most important immune cell populations in the central nervous system and peripheral immune system, respectively, were essential in the pathophysiology of ICH [1, 3]. Therefore, we next focused on analyzing the transcriptional profiles of these two cell types and investigated the crosstalk between central and peripheral immune cells during ICH progression. In addition, the number of B cells was too small for further bioinformatic analyses, so we excluded them from subsequent analyses.

### Single-cell RNA sequencing reveals the complexity of microglia states during ICH progression

**Fig. 2:**
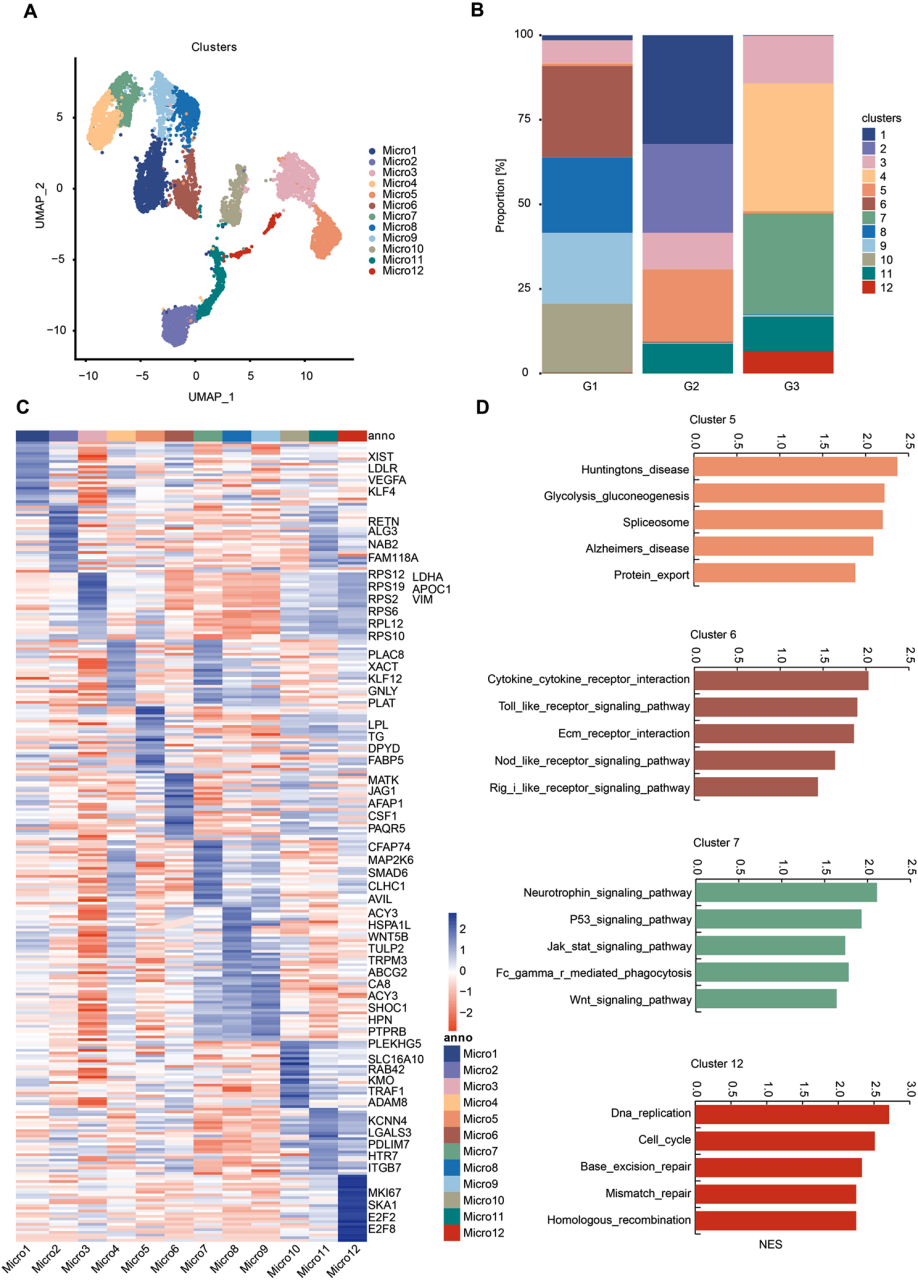
Microglia states have diverse gene expression and biological pathway correlates. **(A)** UMAP of unbiased clustering on the cells from the four sorted clusters (5, 6, 7, and 13, shown in Fig. 1E) meeting criteria for microglia from the 9-sample dataset contains 12 microglia clusters. **(B)** Proportions of all microglia subclusters among three groups. **(C)** Differential expression analysis comparing each cluster to others demonstrates distinct gene expression profiles. The top 25 genes from each cluster are displayed with gene names annotated on the right. **(D)** GSEA analysis of genes that differentiate each cluster from cluster 9 (‘homeostatic microglia’) suggests distinct biological pathways. Permutation-based FDR was used for multiple testing of significance

**Fig. 3:**
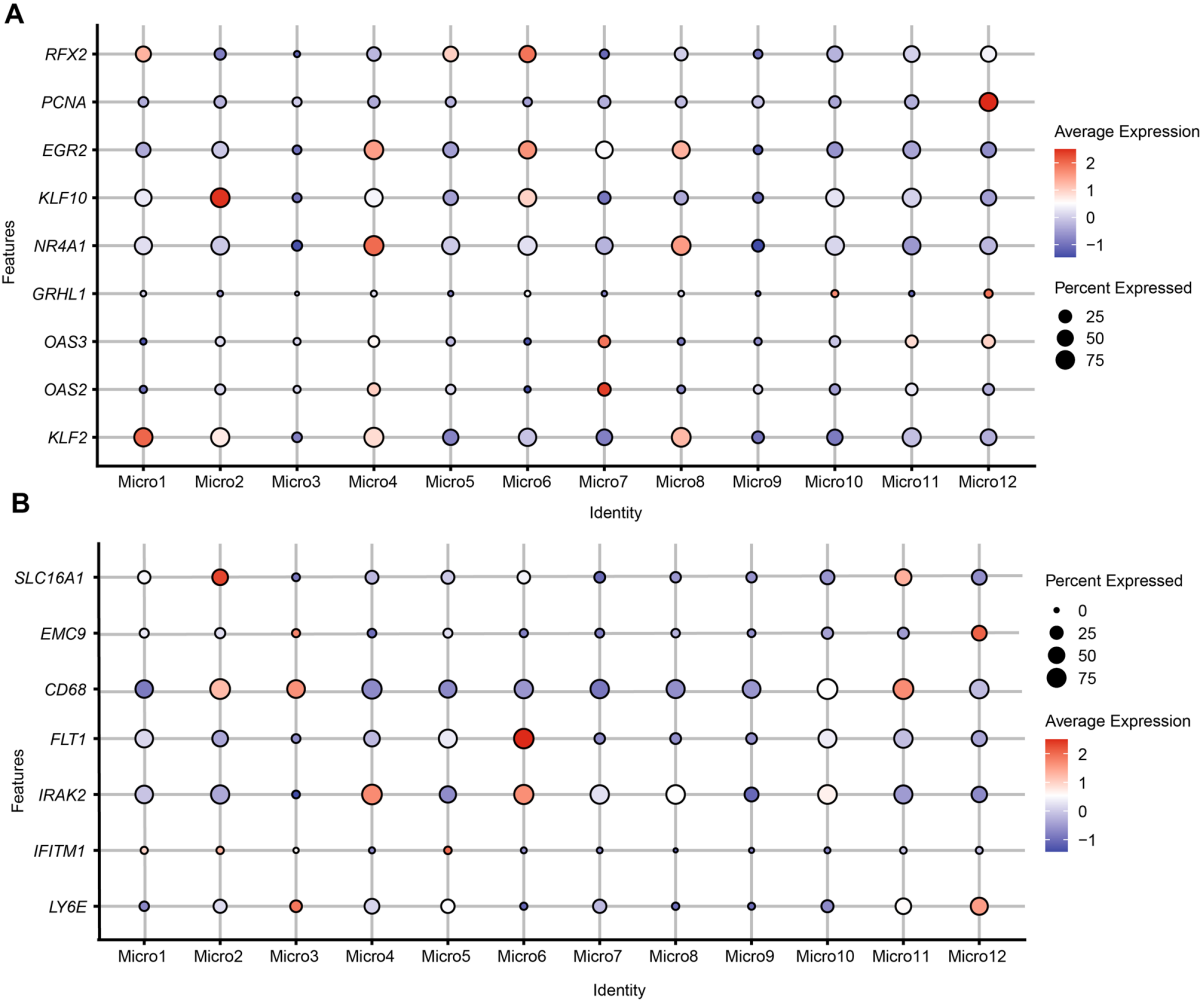
Identifying potential functional marker genes for the microglial clusters. **(A)** Microglial clusters are visualized in columns, and rows represent selected transcription regulators that are differentially expressed in specific clusters. As revealed in the key code at the right of the panel, the size of each dot represents the fraction of cells in a given cluster in which the gene was detected, and the color of the dot represents the average expression levels for the cells belonging to that cluster. **(B)** Representative membrane-associated proteins (y-axis) with the percent of cells that express a gene (size of the dot) in each subcluster (distributed along the x-axis) and the average expression level (color intensity) are shown for microglial subtypes

As a result of cluster analysis of the 8353 microglia-like cells, 12 clusters were identified (Fig. 2A) characterized by differentially expressed genes (DEGs). We determined the enrichment of biological pathways in each cluster via gene set enrichment analysis (GSEA) (Fig. 2D). There was almost no overlap in the DEGs defining each cluster, supporting the unique nature of each microglia cluster (Fig. 2D, Supplementary file: Fig. S1). First, we identified annotated clusters of microglia phenotypically similar to those previously described in the human brain. Cluster-enriched sets of transcriptional regulators and transcription factors were observed in some clusters (1, 2, 4, 5, 6, 7, 8, 10, 11, and 12) but not in the others (3 and 9) (Fig. 3A) [12]. Besides, some selected cell-surface marker genes were not observed in cluster 9 (Fig. 3B). The absence of detectable unique cell-surface markers and on-off transcription factors among clusters 3 or 9 may represent homeostatic microglia, whereas the other clusters differed from them through the upregulation of specific genes. As an additional finding, we identified cluster 9 as an enriched cluster for homeostatic genes, owing to its high levels of P2RY12 and CX3CR1 expression (Supplementary file: Fig. S2A, Supplementary file: Fig. S3A) [13, 14, 21, 22]. However, the higher expression of homeostatic markers was not found in cluster 3 (Supplementary file: Fig. S2A). Therefore, we annotated cluster 9 as homeostatic microglia (HM) cluster. HM was established as a comparative basis for evaluating DEGs from other microglia clusters (Supplementary file: Table 2), referring to the approach in previous literature [12, 21, 22].

Initially, we identified annotated clusters of microglia phenotypically similar to those previously characterized in the human brain. Notably, Cluster Micro3 was characterized by the downregulation of homeostatic genes (Supplementary file: Fig. S3A), such as checkpoint genes (*TMEM119 and CX3CR1*) and purinergic receptors (*P2RY12*), and by the upregulation of encoding many metabolic genes (*APOC1, VIM, LDHA, RPS2, RPS6, RPS10, RPS19, and RPL12*), predominantly ribosomal subunits genes (Fig. 2C). In summary, cluster Micro3 reflects a degenerative phenotype of microglia, consistent with the responses of microglia to aging. Remarkably, Cluster Micro5 predominantly expressed genes characteristic of disease-associated microglia (DAMs), such as metabolic genes *LPL* and *FABP5* (Fig. 2C) [23]. DAM subtype is novel microglia associated with neurodegenerative diseases such as Alzheimer’s [13]. The pathway analysis of the Micro5 genes highlighted associations with “Alzheimer’s disease” and “Huntington’s disease” (Fig. 2D, Supplementary file: Fig. S1B). Hence, we annotated this cluster as “DAM-like” microglia. Cluster Micro6 and Micro10 were defined by genes and pathways involved in canonical inflammatory phenotype. GSEA indicated that these two clusters were enriched in Toll-like receptor (TLR) signaling, Nod-like receptor (NLR) signaling, and chemokine signaling pathway, suggesting inflammatory responses of downstream effectors to stimuli (Fig. 2D, Supplementary file: Fig. S1A). Cluster Micro11 was characterized by anti-inflammatory and repair-related genes (*HTR7*, *PDLIM7*, and *LGALS3*) and proinflammatory genes (*KCNN4* and *ITGB7*) [24–26], indicating that this cluster was an intermediate state in the polarization of microglia (Fig. 2C). Cluster Micro12 was defined by expression of genes involved in DNA repair and cell cycle regulation, including *MKI67*, *SKA1*, *E2F2* and *E2F8* (Fig. 2C). Furthermore, Micro12 was enriched for pathways involved in DNA replication and the cell cycle (Fig. 1D, Supplementary file: Fig. S1B). Micro1 was defined by genes (*XIST*, *VEGFA*, *KLF4*) involved in microglial M1 polarization (Fig. 2C) [27–30]. Moreover, we found that cluster Micro1 was between HM cluster (Micro9) and canonical inflammatory phenotype (Micro6), suggesting that this cluster could be the intermediate transition status from HM microglia to proinflammatory microglia.

Subsequently, we identified four microglial clusters—Micro2, Micro4, Micro7, and Micro8—that had not previously been characterized in human brain studies. These subclusters were distinguished by the enrichment for the pathway of DEGs relative to HM microglia. The significant DEGs of Micro7 were involved in the neurotrophin signaling pathway and Fc gamma receptor-mediated phagocytosis (Fig. 2D, Supplementary file: Fig. S1B). Micro4 showed gene enrichment for complement and coagulation cascades and the PPAR signaling pathway (Supplementary file: Fig. S1A), partially sharing a subset of DEGs with Micro7 (Fig. 2C). The activation of the PPAR signaling pathway has been demonstrated to attenuate proinflammatory responses and increase neurotrophic factors in patients with ICH. Accordingly, we annotated these two clusters as tissue repair phenotypes. Additionally, the pathway significantly enriched Micro8 in antigen processing and presentation, suggesting this subcluster of microglia is active in antigen processing and presentation for immune response (Supplementary file: Fig. S1A). Furthermore, the pathways enriched in Micro2 confirm the relative increase of genes involved in oxidative phosphorylation and glycolysis/gluconeogenesis and decreased chemokine and endocytosis genes (Supplementary file: Fig. S1A). This finding coincides with previous studies that persistent glycolysis exerts adverse effects on microglial functions: the activation of glycolytic metabolism impairs phagocytosis and chemotaxis of microglia [31, 32]. Overall, while future research will likely refine our understanding of microglial subtypes, our study significantly advances the knowledge of microglial heterogeneity in human PHE tissue.

### Microglia subclusters predominantly exhibit proinflammatory phenotypes after ICH

Previous studies of single-cell transcriptomics have shown diverse subclusters of microglia, which are considered to reflect their different functions. In this study, we employed scRNA-seq to investigate the biological pathways present in microglia within PHE tissue following ICH. We observed that common microglial marker genes such as *AIF1, TREM2*, and *CSF1R* were widely expressed across all microglial subclusters (Supplementary file: Fig. S3A). However, other specific marker genes of microglia (*ITGAM*, *P2RY12*, and *CX3CR1*) indicated differential expression across clusters (Supplementary file: Fig. S3A). Interestingly, the transcriptome of PHE tissue was almost dominated by proinflammatory pathways (Supplementary file: Figs. S3A-B). Our findings indicated a lack of significant activation of anti-inflammatory pathways within the first 48 h post-ICH (Supplementary file: Fig. S3B). Proinflammatory genes such as *CCL2, CCL4*, and *IL1B* were among the most prevalently expressed cytokine and chemokine genes in ICH-associated microglia (Supplementary file: Fig. S3A-B). ICH microglial clusters 1, 3, 5, 6, 10, and 11 were characterized by high gene expression levels of *HLA-DQA1*, *HLA-DPB1*, and *HLA-DRA* and low gene expression levels of *P2RY12* and *CX3CR1* (Supplementary file: Fig. S3A), suggesting their involvement in the primary immune response to ICH. Complement pathway-related genes (*C3, C1QB, and C1QC*) also maintained high levels of expression in all microglial cell clusters (Supplementary file: Fig. S3B). Overall, most microglial clusters displayed proinflammatory phenotype within 48 h of ICH, further corroborating the notion that a proinflammatory response is a key pathogenic mechanism in ICH.

Purinergic receptor P2RY12, the cell-surface proteins of microglia, play key roles in mediating neuroinflammatory responses [33]. Our results indicated reduced expression of the *P2RY12* gene in microglia clusters that had higher IL1B expression levels (Supplementary file: Fig. S2A). This finding was consistent with the previous studies that the expression of P2RY12 was gradually decreased accompanied by microglia activation following inflammatory stimulation [34]. Further analysis was performed by comparing the differentially expressed genes in IL1B-expressing clusters (cluster 6 and cluster 10) with those in P2RY12-expressing clusters (cluster 7 and cluster 9). A significant difference in gene expression was found between IL1B-expressing microglia and P2RY12-expressing microglia, with 882 genes notably downregulated and 415 genes notably upregulated (adjusted *P* value < 0.05, log2 (fold change) > 1.5) (Supplementary file: Fig. S2B, Supplementary file: Table 3). A significant increase in chemokine and pro-inflammatory cytokines was observed in microglial cluster cells expressing IL1B (Supplementary file: Fig. S2C). Additionally, CX3CR1 expression was also higher in cluster cells expressing P2RY12 than in cluster cells expressing IL1B (Supplementary file: Fig. S2B). In addition, Gene Ontology term enrichment analysis indicated genes enriched for protein binding, extracellular exosome, focal adhesion, cytokine-mediated signaling pathway, and inflammatory response (Supplementary file: Fig. S2D). Furthermore, the Kyoto Encyclopedia of Genes and Genomes term enrichment analysis suggested genes enriched for apoptosis, NF − kappa B signaling pathway, IL − 17 signaling pathway, and Toll − like receptor signaling pathway (Supplementary file: Fig. S2E). According to DEGs analysis, pro-inflammatory microglia expressing IL1B are structurally and functionally different from those expressing P2RY12. According to these findings, as well as previous studies, PHE tissue removed from patients with ICH contains an immune pathogenic microenvironment that attracts and induces non-specific and specific immunity rapidly. Hence, we emphasized on the characterization of immune cells infiltrating in PHE tissues.

### Microglia cluster-specific transcription factor regulatory networks

**Fig. 4:**
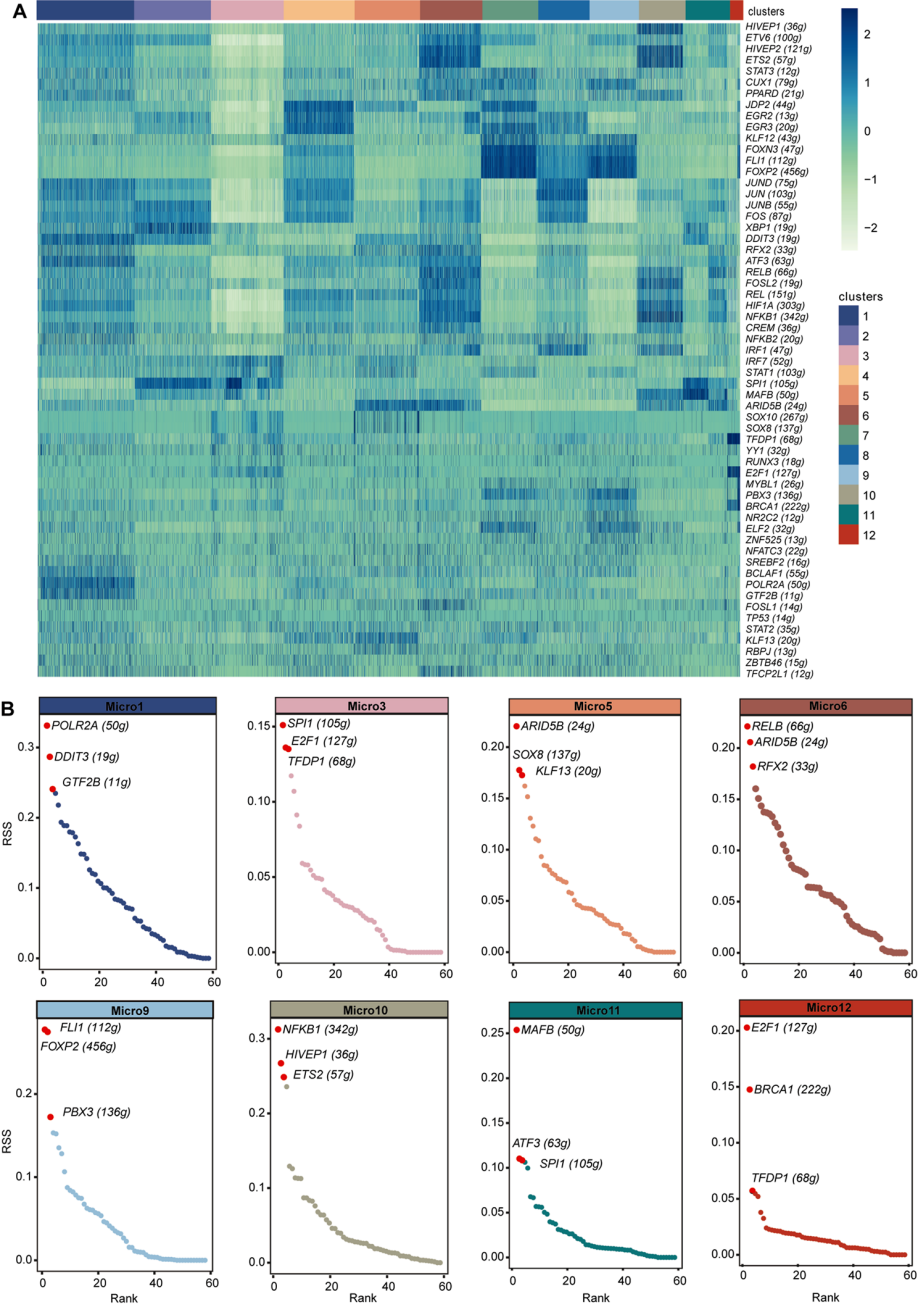
Transcription factor regulatory networks are specific to microglia phenotypes **(A)** SCENIC workflow identified transcription factor-regulated networks associated with different phenotypic clusters of microglia. Heatmap of each microglia subtype’s inferred regulon activity score (RAS) in cell levels. **(B)** Ranking plot of regulon specificity score (RSS). The higher RSS of the regulon may be specific to the subtypes

To explore the regulatory networks of the microglia clusters in the dataset, we also applied SCENIC analysis to identify the top transcription factor-driven networks (regulons) controlling gene expression in each of these 12 microglia clusters (Fig. 4A, Supplementary file: Fig. S4A). Each microglia cluster was characterized by a specific set of regulons (Fig. 4B). This supports the theory that transcriptional regulation mechanisms are key determinants of the unique gene expression profiles observed in each microglia cluster. For example, Micro1 showed higher activity levels of *POLR2A*, *NFKB2*, *GTF2B*, and *BCLAF1* (Fig. 4A, Supplementary file: Fig. S4A). Micro3 showed higher activity levels of *SOX8*, *SOX10*, *IRF7*, and *STAT1* (Fig. 4A, Supplementary file: Fig. S4A); The activity of transcription factors, such as *MAFB*, *SPI1*, *DDIT3*, and *XBP1*, was higher in Micro11, while high activity levels of *E2F1*, *TFDP1*, and *BRCA1* were associated with Micro12 (Fig. 4A, Supplementary file: Fig. S4A). Furthermore, our analysis revealed that *MAFB*, a regulon governed by transcription factors commonly linked with the anti-inflammatory polarization of human microglia, was prominently featured in the Micro11 cluster (Fig. 4B, Supplementary file: Fig. S4C). This is consistent with the finding that these cells experience a phenotypic polarization of microglia of M2. In this study, the *NFKB1* regulon, associated with canonical inflammatory responses, was identified in Micro10 (Fig. 4B, Supplementary file: Fig. S4C). Conversely, Micro6 exhibited the *RELB* regulon, linked to non-canonical inflammatory responses (Fig. 4B, Supplementary file: Fig. S4C). In Micro9, the high specificity of *FOXP2* (Supplementary file: Figs. S4C), a regulon unique to human microglia and crucial for brain development, was observed, aligning with previous research identifying this subtype as an HM cluster [35]. The top three regulons in other microglia clusters also showed distinct variations (Fig. 4B, Supplementary file: Figs. S4B-C). These inferred transcription factor regulons provide insight into the diversity and difference within microglial clusters, suggesting novel potential regulatory targets for future research.

### The SPP1 signaling pathway was the fundamental bridge to self-communication among microglia subclusters

**Fig. 5:**
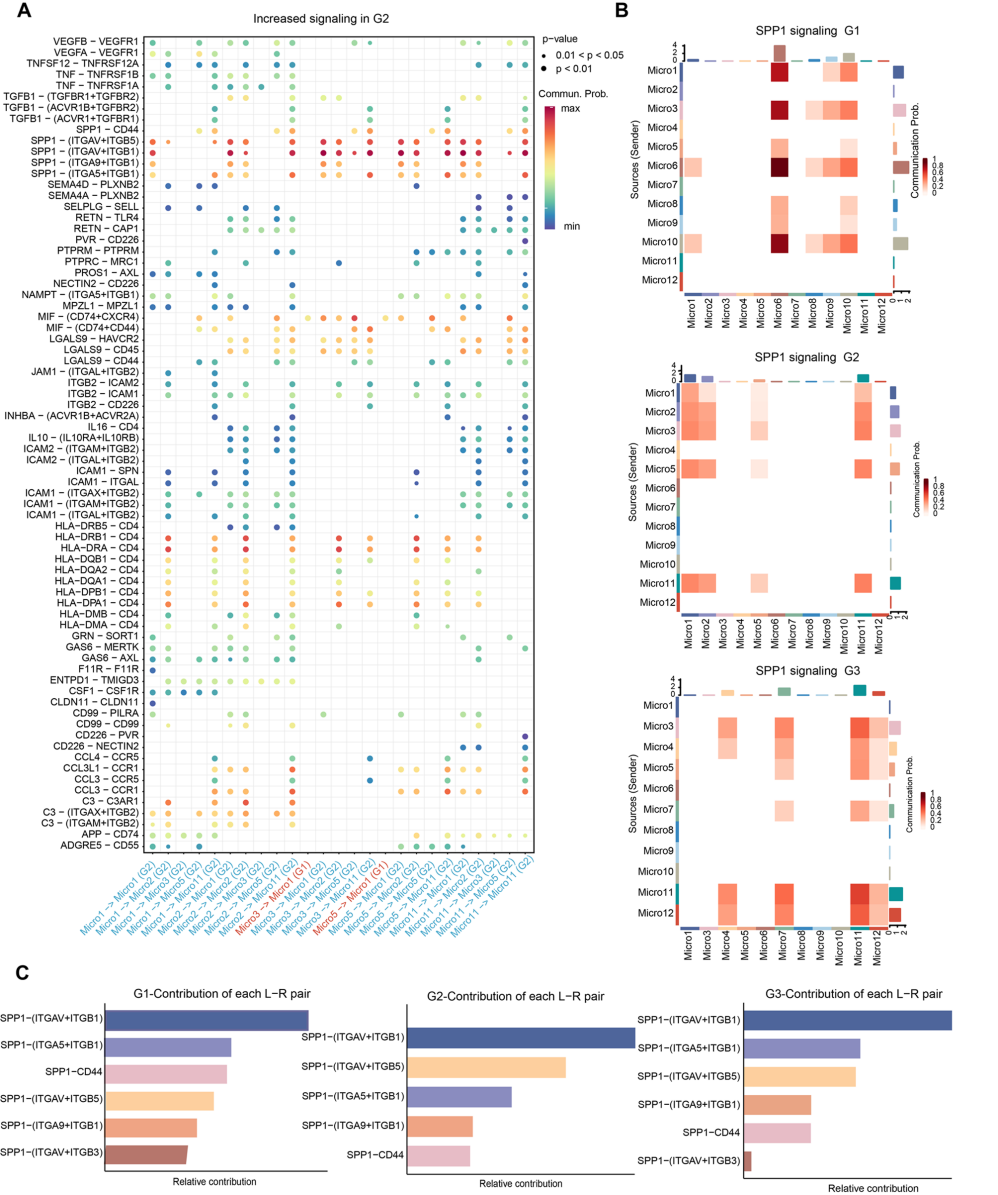
Signaling changes of microglia subcluster during PHE tissue progression. **(A)** Cell ligand-receptor inference analysis of microglial subtypes during PHE tissue progression (G2 vs. G1). **(B)** Heatmaps demonstrate the differences in crosstalk strength of the *SPP1* signaling pathway in microglial subclusters between different groups (G1, G2, and G3). **(C) **The contribution of inferred ligand-receptor pairs in *SPP1* signaling between different groups (G1, G2, and G3)

Along with the PHE progression, microglial subtypes also changed accordingly. The use of cell-cell communication networks between microglia subpopulations could contribute to a better characterization of microglia function. Interestingly, the interaction strength of the *SPP1* pathway increased gradually with the progression of PHE (Fig. 5A, Supplementary file: Fig. S5). Moreover, the *SPP1* pathway exhibited the strongest interaction strength, irrespective of incoming or outgoing signaling pathways (Fig. 5B). It indicates that the *SPP1* signaling pathway could be responsible for self-communication between microglia subclusters. Regarding the incoming signaling, *SPP1* was emitted by different microglial subclusters at different stages. At post-ICH in G1, G2, and G3, the strongest *SPP1* signaling cell types were Micro6, Micro1, and Micro11, respectively (Fig. 5B). Regarding outgoing *SPP1* signaling, the Micro6 subtype was also strongest at G1 after ICH. In ICH patients at G2 and G3, Micro11 showed strong *SPP1* signals (Fig. 5B). Additionally, we visualized the crosstalk between each microglia subcluster in the *SPP1* signaling pathway. We explored the specific receptor ligands and found that the *SPP1*-( *ITGAV* + *ITGB1*) ligand-receptor pair was the fundamental bridge of self-communication among microglia subclusters (Fig. 5C). Collectively, our findings demonstrated that the signaling pathway of *SPP1* is the fundamental bridge mediating self communication between subclusters of microglia during the progression of ICH.

### Time-dependent transcriptional heterogeneity of neutrophils in the human brain after ICH

**Fig. 6:**
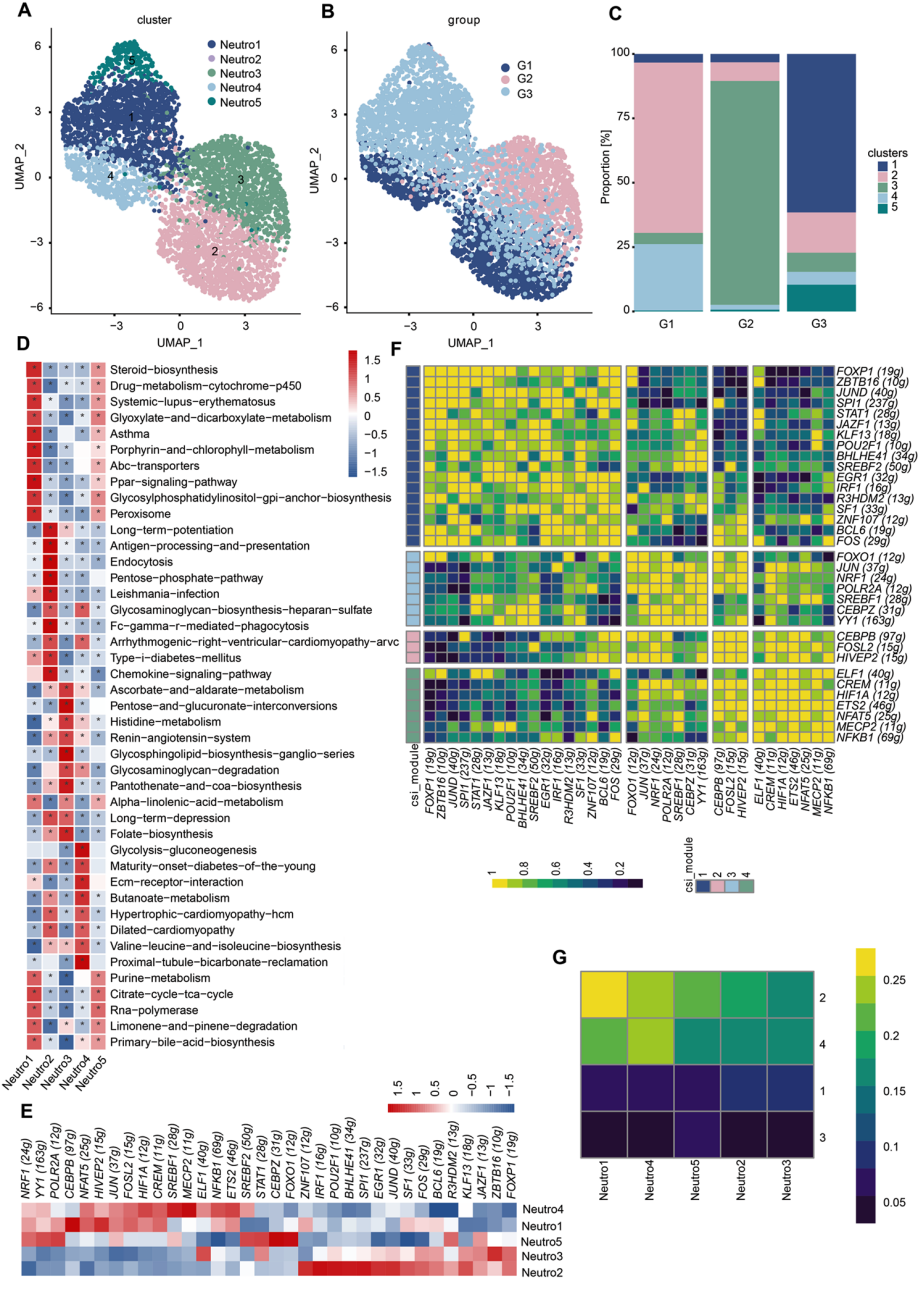
The heterogeneity and transcription factor regulatory networks of neutrophils during PHE progression. **(A)** UMAP of unbiased clustering on the cells for neutrophils from the 9-sample dataset contains five neutrophil clusters. **(B)** Distribution of neutrophils from different groups (G1, G2, and G3) on a UMAP plot. **(C)** Proportions of all neutrophil subclusters among three groups. **(D)** GSVA analysis indicates enriched pathways of each subset of neutrophils. Benjamini and Hochberg (BH) FDR procedure was used for multiple testing of significance. **(E)** Heatmap of each neutrophil subtype’s inferred regulon activity score (RAS) in cluster levels. **(F)** The connection specificity index (CSI) matrix highlights the regulon-to-regulon correlation across all neutrophil subtypes from different groups. Hierarchical clustering of regulons identifies four distinct regulon modules. The heatmap shows the regulation activity of each module. **(G)** Heatmap of the CSI matrix across all neutrophil subtypes. The color key from blue to yellow indicates the activity levels from low to high

To analyze neutrophil transcriptional heterogeneity during ICH progression, we performed a multiplexed time series of scRNA-seq analyses combining transcriptomics. By comparing the gene expression patterns of all neutrophils at every time points, we depicted five different transcriptional cell clusters in PHE tissue after ICH, exhibiting a time-independent appearance. Due to the fact that the number of cells sampled at each time point (G1: 1312 cells; G2: 1132 cells; G3: 2109 cells) cannot reflect the true level of neutrophils in ICH PHE tissues (Fig. 6B), we calculated the proportion represented by each cluster at different time points (Fig. 6C). Surprisingly, we found that cluster Neutro1 (*MECMP1, TNFAIP3, IER3, TANK*) increased to reach its highest level at G3 (*P <* 0.05, G3 vs. G1 and G3 vs. G2, Supplementary file: Table 4). The majority of neutrophils at G1 (68.6%) segregated in cluster Neutro2 (*CFD*, *CDKN2D*, *HSPA1B*, *S100A4*, *D100A6*, *S100A9*, *S100A12*), a profile reduced by half at later time points (< 30% at G2 and G3). At G2, Neutro3 cells (*FOLR3, SLC8A1, AOAH, SYNE2*; 86.9% of total neutrophils) were predominant. Cluster Neutro5 (*DPYD*, *KIFC3*, *BMP2K*, *ABHD5*, *SPDYA*) was present at all time points and its levels significantly increased from 0.4% of cells at grade 1 to 10.4% at G3. Finally, cluster Neutro4 was characterized by highly specific expression of some transcripts (*PLNA*, *PFN1*, *HMGA1*), and represented 26.8%, 1.9% and 5.0% of all neutrophils at G1, 2 and 3 post-ICH, respectively (Fig. 6C, Supplementary file: Fig. S6A).

The unique gene signatures and the top 20 significantly DEGs of each neutrophil subcluster were delineated (Supplementary file: Fig. S6A). In addition, the Gene Set Variation Analysis (GSVA) was performed to functionally annotate the neutrophil subcluster (Fig. 6D). Neutro1 showed gene enrichment for steroid biosynthesis, ABC transporters, and PPAR signaling pathway, partially sharing a subset of significantly differentially expressed genes with Neutro5 (Fig. 6D). Neutro2 was characterized by the chemokine signaling pathway and antigen processing and presentation (Fig. 6D), indicating this subtype of cells is active in antigen processing and presentation for immune response. The significantly DEGs of Neutro3 were involved in the folate biosynthesis and the metabolism-related pathways including histidine metabolism, alpha-linolenic acid metabolism, and ascorbate metabolism (Fig. 6D). Neutro4 exhibited higher expression of genes associated with glycolysis and gluconeogenesis, and extracellular matrix (ECM)-receptor interaction (Fig. 6D). Neutrophils can carry preexisting matrix from nearby tissue to reestablish new ECM scaffold in the early stages of tissue repair, suggesting that these cells may represent a repair phenotype [36]. Furthermore, Neutro5 was defined by the expression of genes involved in RNA polymerase and TCA cycle (Fig. 6D).

### Neutrophil cluster-specific transcription factor regulatory networks

We further employed SCENIC analysis to characterize the underlying molecular mechanisms driving the differentiation of different neutrophil phenotypes. A different transcription factor network was predicted to be expressed by neutrophils from different subtypes in this analysis. For instance, *CEBPB* and *HIVEP2* regulons were upregulated in Neutro1 (Fig. 6E). Consistent with the findings that the *CEBPB* regulon is critical for the emergency granulopoietic response, this mechanism fulfills the increased demand for neutrophils during the innate immune response to inflammation [37]. Additionally, there was a notable increase in the Neutro1 population, peaking at G3 (Fig. 6C). This suggests a substantial consumption of neutrophils at this stage, prompting the hematopoietic system to respond to the heightened demand through emergency granulopoiesis quickly. Neutro2 also upregulated networks driven by *ZNF107*, *IRF1*, and *SPI1* (Fig. 6E). In the Neutro3 cluster, there was a noticeable predominance of the activity of regulons, particularly those linked to *ZBTB16* and *ELF1* (Fig. 6E). Some regulons showed preferential activity in Neutro4 (*MECP2*, *SREBF1*, *HIF1A*; Fig. 6E). Neutro5 showed highly active transcription factors related to cell proliferation (*FOXO1*, *CEBPZ*, *YY1*; Fig. 6E). By calculating the connection specificity index (CSI), we obtained a regulatory network composed of four modules and 34 regulons (Fig. 6F). Transcription factors of CEBPB/FOSL2/HIVEP2 in module 2 displayed the strongest activity in Neutro1 (Fig. 6F-G) and were related to emergency granulopoiesis.

### Trajectory analysis of neutrophils in PHE tissue during ICH progression

**Fig. 7:**
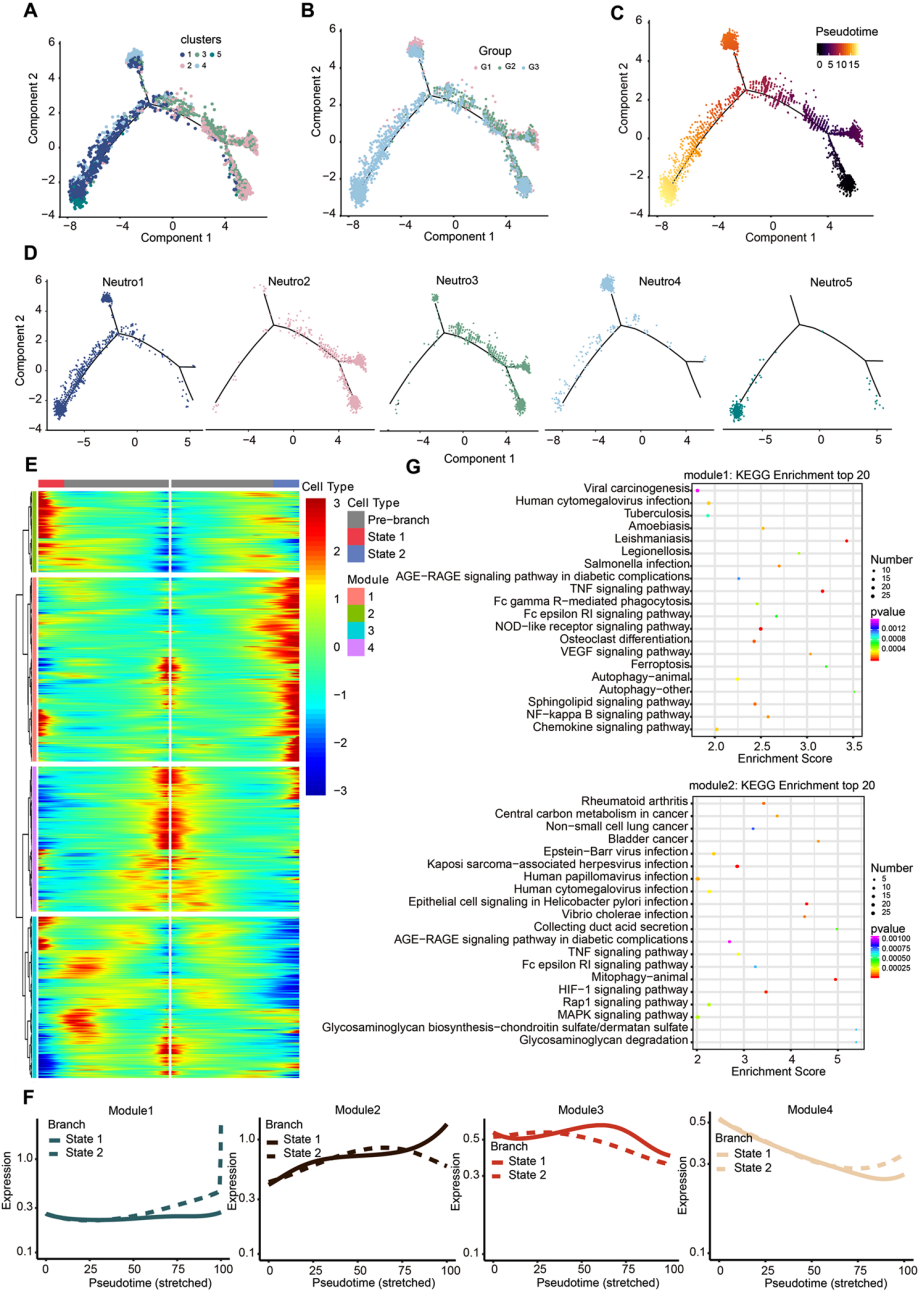
Exploring the transition of neutrophils based on pseudo-time analysis using Monocle2. **(A)** Trajectory of five clusters along pseudo-time in a two-dimensional state-space defined by Monocle2. Each point corresponds to a single cell, and each color represents a neutrophil cluster (q-val < 0.01, BH method) **(B)** The pseudo-time trajectory plots demonstrate the sample distribution along the trajectory. The dot color represents the group. **(C)** The developmental pseudo-time of neutrophils was inferred by Monocle analysis. The dark to bright color key indicates cell differentiation from early to late. **(D)** The pseudo-time trajectory plots demonstrate the sample distribution along the trajectory. Each dot color represents a neutrophil cluster. **(E)** The heatmap displays the significantly differential expression genes during the trajectory. The blue-to-red color key indicates low to high relative expression levels. **(F)** Averaged expression patterns of gene sets from four modules along pseudo-time. **(G)** Enriched KEGG terms for gene sets from two modules (modules 1 and 2) were represented on the left side

In order to explore the dynamic transition of neutrophils from peripheral blood to PHE tissue, we constructed a pseudotime map of the neutrophil state trajectory using monocle2 (Fig. 7A and B, and 7D). Neutrophils are also known as short-lived cells that can mobilize rapidly from the bone marrow in response to tissue damage [38]. Accordingly, we performed a ‘bone marrow proximity score’ of neutrophils based on the expression of a set of genes previously characterized in transcriptome analyses of neutrophils at different stages [39]. Cluster Neutro2 (mainly in G1) had the highest BM proximity score (*p* < 0.0001 versus all other clusters) (Supplementary file: Fig. S6B).

In our analysis, the progression trajectory of neutrophils was established, starting with Neutro2 (Fig. 7C). This was followed by Neutro3, serving as a transitional state between Neutro2 and Neutro4, then moving through an intermediate infiltrating phase represented by Neutro1, and culminating in the terminally differentiated states of Neutro4 and Neutro5. Further examination of the single-cell transcriptomes of neutrophils along this trajectory identified 4043 significantly altered genes, classified into four distinct expression patterns (Fig. 7E-F): Module 1 included genes that showed increased expression levels along one trajectory. Pathway enrichment analysis suggested that these genes participated in the chemokine signaling pathway, NOD-like receptor signaling pathway, NF-kappa B signaling pathway, and TNF signaling pathway (Fig. 7G). Module 2 contained genes activated in the later stages of another trajectory, with enrichment in mitophagy and HIF-1 signaling pathways (Fig. 7G). Additionally, the genes in module 3 were associated with ribosome function, Fc gamma R-mediated phagocytosis, endocytosis, regulation of actin cytoskeleton, and leukocyte transendothelial migration (Supplementary file: Fig. S6C). Finally, module 4 genes, upregulated in the early stages, were associated with necroptosis, apoptosis, and cellular senescence (Supplementary file: Fig. S6C).

### OPN-mediated microglia-monocyte interaction was essential for the crosstalk between central and peripheral immune cells

**Fig. 8:**
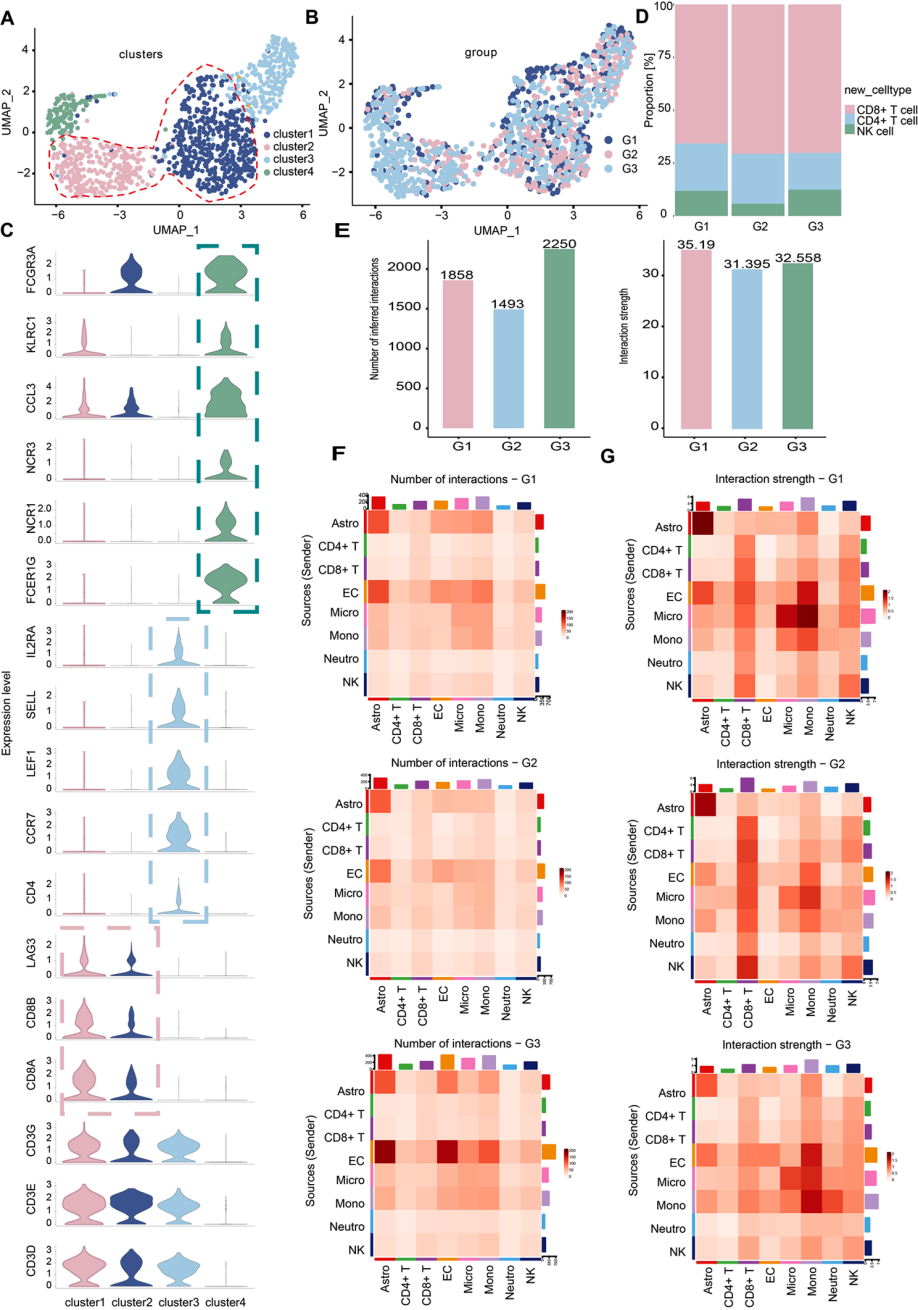
Intercellular ligand-receptor prediction among immune cells revealed by CellChat analysis. **(A)** The UMAP plot displays four subclusters of NK/T cells. **(B)** Distribution of NK/T cells from different groups (G1, G2, and G3) on a UMAP plot. **(C)** Representative cell type marker genes (y-axis) in each cluster (distributed along the x-axis) are shown for CD8 + T cells (*CD8A*, *CD8B*, *LAG3*), CD4 + T cell (*CD4*, *CCR7*, *LEF1*, *SELL*, *IL2RA*), and NK cell (*FCER1G*, *NCR1*, *NCR3*, *CCL3*, *KLRC1*, *FCGR3A*). **(D)** Proportions of CD4 + T cell, CD8 + T cell, and NK cell among three groups. **(E)** Bar plot showing the number and strength of intercellular interactions during PHE progression. Heatmaps of differential number (**F**) and strength (**G**) of intercellular interactions during PHE progression

**Fig. 9:**
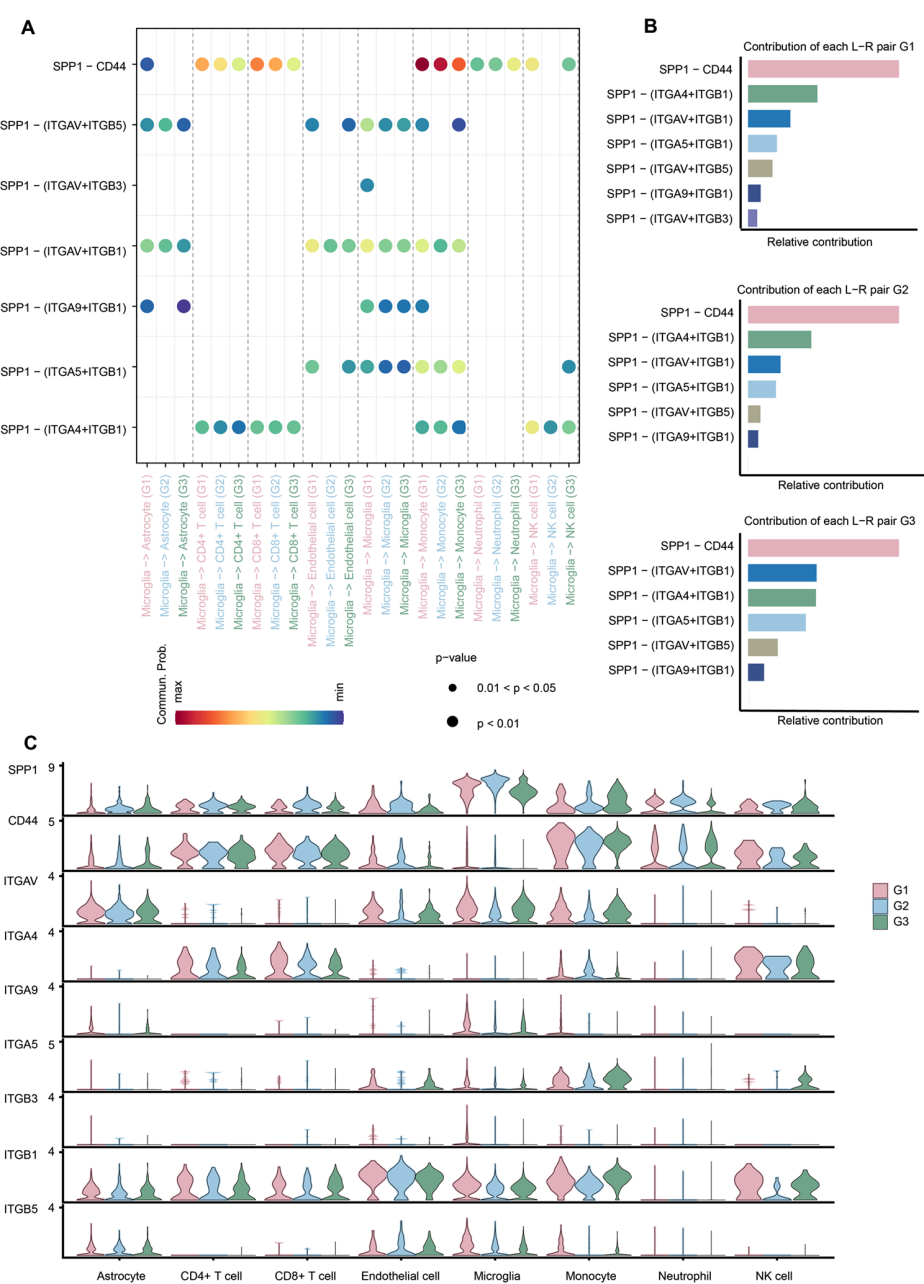
Cell ligand-receptor inference analysis of immune cells during PHE progression. **(A)** Bubble plot (excerpted in the red dotted box in Supplementary file: Fig. S7) of the significantly differentially expressed ligand-receptor pairs during PHE progression. Dot color reflects communication probabilities, and the dot size represents computed p-values. Empty space means the communication probability is zero. P-values are computed from a two-sided permutation test. **(B)** The contribution of inferred ligand-receptor pairs in *SPP1* signaling between groups (G1, G2, and G3). **(C)** Violin plots of expression distribution of signaling pathway-related genes

We previously discovered a mixed cluster (cluster 11) by immune cell analysis (Fig. 1E). Cluster 11 clearly showed *CD3D*, *CD3E*, *NKG7*, *GZMA*, and *GZMB* gene expression, indicating doublets of T cells and NK cells (Fig. 1E). Cluster NK/T cell was present at all time points and its levels gradually increased during ICH progression (Fig. 1D and Fig. 8B). We re-clustered cluster 11 doublet cells using major lineage gene expression markers and obtained four clusters (Fig. 8A). Cluster phenotypes were identified using gene expression levels (Fig. 8C). A CD8 + T cell cluster (*CD8A*, *CD8B*, *LAG3*; cluster 1 and cluster 2) was the main cluster found in PHE tissue (Fig. 8C-D). We also observed CD4 + T cells (*CD4*, *CCR7*, *LEF1*, *SELL*, *IL2RA*; cluster 4) and NK cells (*FCER1G*, *NCR1*, *NCR3*, *CCL3*, *KLRC1*, *FCGR3A*; cluster 3) (Fig. 8C). By inferring the paired ligand-receptor pairs based on CellChat analysis, we first depicted the overall connectivity patterns between peripheral immune cells and central immune cells in PHE. The number of cell-cell interactions between immune cells changed significantly in the context of the progression of ICH (Fig. 8E-F). However, the strength interaction involving microglia, monocytes, and CD8 + T cells is consistently higher in the progression of ICH (Fig. 8G). Intriguingly, monocytes exhibited more extensive communications with microglia than other immune cell types apart from CD8 + T cells and NK cells (Fig. 8G). We then extracted highly expressed interactions engaging microglia during ICH progression and uncovered underlying interactions with monocytes (Fig. 9A, Supplementary file: Fig. S7). Notably, we found interactions between *SPP1*, which encodes the pleiotropic cytokine OPN, and *CD44*, *ITGAV*, *ITGA4*, *ITGA5*, *ITGA9*, *ITGB1*, *ITGB3*, and *ITGB5*, which encode the OPN receptor, were prominent during microglia-monocyte interactions (Fig. 9A). Furthermore, the pair of OPN-CD44 stands out among all interaction pairs that mediate the crosstalk between microglia and monocytes and displays the highest score (Fig. 9A-B, Supplementary file: Fig. S7). Additionally, we found that the *SPP1* gene was primarily expressed in the microglia rather than in other immune cells. In contrast, the *CD44* gene was mainly expressed in the monocytes (Fig. 9C). This finding suggests that microglia-secreted OPN could regulate the immune environment of PHE by interacting with CD44 on monocytes. In summary, these findings indicate that OPN-mediated microglia-monocyte interaction is essential for communicating between central and peripheral immune cells.

### Immunofluorescence staining

**Fig. 10:**
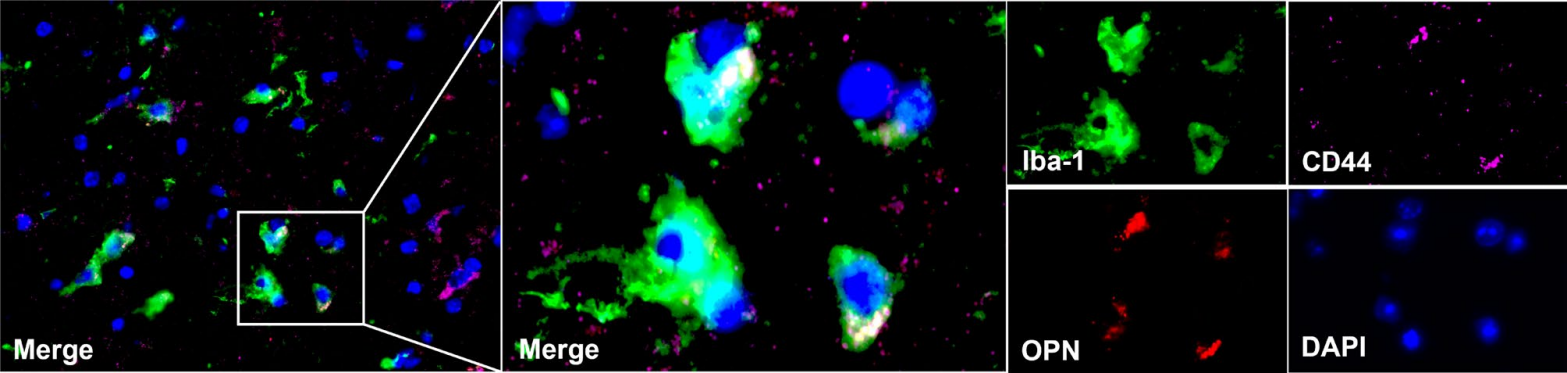
Co expression of osteopontin and CD44 in the tissue adjacent to cerebral hemorrhage hematoma. The following pictures are all taken under the Zeiss Z2 laser confocal microscope, showing co-staining and individual staining of several molecules, respectively. The surface markers Iba-1 of microglia, CD44 of monocytes, and OPN are labeled with green, red, and pink fluorescent signals respectively, while blue is the labeling of the nucleus. Microglia are surrounded by a large amount of OPN, and there is a high degree of spatial co localization between OPN and CD44.

To verify our conclusion, we extrally collected a group of adjacent hematoma tissues from patients with ICH for immunofluorescence experiments. Iba-1 (surface marker of microglia), CD44 and OPN were labeled with different colors of fluorescence for immunofluorescence co staining. Afterwards, we used a Zeiss Imager Z2 confocal microscope to observe the stained tissue sections, such image was captured under a microscope: co-localization existing between OPN and microglia (Iba-1), and highly spatially close to CD44 (Fig. 10). This also coincides with the conclusion drawn from our previous analysis: (1) Osteopontin is secreted by microglia; (2) OPN as a mediator participates in the activation of microglia and CD44 cells, especially monocytes.

## Discussion

In this study, we characterized the immune cell landscape of PHE tissue in patients with ICH, uncovering the predominant proinflammatory microenvironment shaped by microglia. Using SCENIC analysis, we observed that each cluster was driven by different transcription factors, further supporting the functional diversity of clusters. Trajectory inference analysis revealed transitions and putative relationships between neutrophil phenotypes. Cell-cell communication networks analysis indicated that the *SPP1* signaling pathway was the fundamental bridge of self-communication among microglia subclusters. Furthermore, we identified that microglia-derived OPN was an essential mediator in the communication between microglia and monocytes.

Our study used scRNA-seq analyses to accurately distinguish between immune and non-immune cells in the human brain, identifying their specific types and biological functions. Our study suggested microglia as a major contributor to inducing a proinflammatory immune microenvironment, mainly through producing proinflammatory mediators and reducing microglial purinergic receptor-mediated signaling. Recent studies have shown that the expression level of P2RY12 in microglia is correlated with the phenotype of microglia [34]. Specifically, it is less expressed by activated proinflammatory microglia, and highly expressed by activated non-inflammatory microglia [40, 41]. Reductions in P2RY12-expressing microglia indicated the presence of a broad proinflammatory milieu in PHE tissue. Furthermore, it has been demonstrated in numerous preclinical studies and clinical trials that modulating microglial polarization (M1/M2) can reduce inflammation and thus exert a protective effect in ICH [4, 42]. However, none of the immunomodulators that targeting neuroinflammation of PHE tissue have been applied in clinical practice. Recently, there has been increasing evidence that the M1/ M2 dichotomy of microglial polarization has been oversimplified, highlighting the need for unbiased high-throughput methods to comprehensively investigate microglial heterogeneity [43]. A single-cell transcriptome study identified nine microglial subclusters in human brain samples from neurosurgical procedures, revealing phenotypes beyond the conventional M1/M2-like patterns [44]. Our study did not observe a complete overlap between the microglia subtypes and classical M1 or M2 subtypes after ICH at the single-cell level. Conversely, we identified 12 distinct microglial subclusters in PHE tissue. Similarly, another scRNA-seq study in a mouse ischemic stroke model identified six distinct microglial subclusters; none fully conformed to the M1 or M2 microglia marker gene [45]. Moreover, our SCENIC analysis demonstrated the diversity of the identified microglial clusters and suggested potential regulatory targets for future research, highlighting the complexity and nuance of microglial roles in the ICH context.

As the first activated immune cell cluster, microglia undergo various phenotypic and functional changes depending on the stimuli involved after ICH [46]. Our study has revealed the significance of the OPN-CD44 receptor axis in the interaction between microglia and monocytes. OPN, a multifunctional phosphoglycoprotein, bridges innate and adaptive immune responses under pathological conditions [47]. Previous studies demonstrated that OPN is involved in neuroimmune responses in diverse central nervous system diseases, including ICH, ischemic stroke, and AD [48–50]. Furthermore, recent evidence suggests that OPN is highly expressed by microglia, which plays a significant role in immunomodulatory effects [51, 52]. Cell-cell communication analyses predicted strong interactions between microglia and monocytes through OPN and CD44 receptors. These interactions need to be experimentally validated by a range of in vitro and in vivo investigations in the future. Prior studies indicated that peripheral monocytes are also cellular sources of OPN but with lower expression than microglia [53]. Moreover, recent single-cell studies have noted an increased transcription of *SPP1*, the gene encoding OPN, in monocytes in the brains of mice with AD [50]. Therefore, we speculated that microglia act as initiators of OPN production in monocytes, which could be crucial for monocyte infiltration. However, it is important to note that our study could not determine whether additional OPN-independent mechanisms are involved in the communication between microglia and monocytes.

## Conclusion

In conclusion, we generated the first scRNA-seq data set for immune cells in the human PHE tissue and provided novel insights into post-ICH microglia and neutrophil heterogeneity in the brain. We discovered that the *SPP1* signaling pathway was the fundamental bridge of self-communication among microglia subtypes during ICH progression. Additionally, OPN has been identified as a mediator between monocytes and microglia. Our findings, therefore, are considerable in developing a more comprehensive understanding of immune cell diversity, which will drive the development of immunomodulators targeted neuroinflammation post-ICH. Therefore, our findings are of great significance for a more comprehensive understanding of immune cell diversity, which may contribute to exploit immunomodulators targeted neuroinflammation post-ICH.

### Limitations

There were several limitations in our study. Firstly, the study is limited by a small sample size consisting solely of individuals of Asian descent, potentially restricting the generalizability of the findings. Additionally, the absence of control trials within the experimental group undermines the strength of the conclusions drawn. Thirdly, the inability to fully differentiate between gray and white matter in the sampled tissue, coupled with the reliance solely on immunofluorescence for validation without comparison to a control group, highlights the need for more thorough and rigorous scientific verification. Fourthly, The SCENIC analysis utilizing single-cell transcriptome data serves as a bioinformatics tool for predicting potential activation levels of transcription factors and their regulation of target genes. However, further experimental validation is necessary to confirm the identified transcriptional regulators. Lastly, it is important to note that the brain tissue samples used in this study did not differentiate between white matter and gray matter, and the inherent variability in clinical bio-samples and the dissociation process in scRNA-seq may result in the loss or bias towards certain cell types, rather than providing a comprehensive representation of the cellular composition of the original sample.

### Electronic supplementary material

Below is the link to the electronic supplementary material.

Supplementary Material 1

Supplementary Material 2

Supplementary Material 3

Supplementary Material 4

Supplementary Material 5

Supplementary Material 6

Supplementary Material 7

Supplementary Material 8

Supplementary Material 9

Supplementary Material 10

Supplementary Material 11

Supplementary Material 12

Supplementary Material 13

## Data Availability

Our scRNA-seq data from human brains is available in the form of a browsable platform. The raw data our study is available at the Gene Expression Omnibus under accession code. The accession number for the sequencing data reported in this paper is GEO:GSE 266873. Source data are provided with this paper.

## Abbreviations

BBB: Blood-brain barrier
ICH: Intracerebral hemorrhage
GSEA: Gene Set Enrichment Analysis
GSVA: Gene Set Variation Analysis
OPN: Osteopontin
PHE: Perihematomal edema
SCENIC: Single-cell regulatory network inference and clustering
scRNA-seq: Single-cell RNA sequencing
SPP1: Secreted phosphoprotein-1
UMAP: Uniform manifold approximation and projection
UMI: Unique molecular identifiers
