# Supplementary figures and images for: Single-cell RNA sequencing reveals the evolution of the immune landscape during perihematomal edema progression after intracerebral hemorrhage
Source: J Neuroinflammation. 2024 May 28;21:140. doi: 10.1186/s12974-024-03113-8 (PMC11131315; doi:10.1186/s12974-024-03113-8)

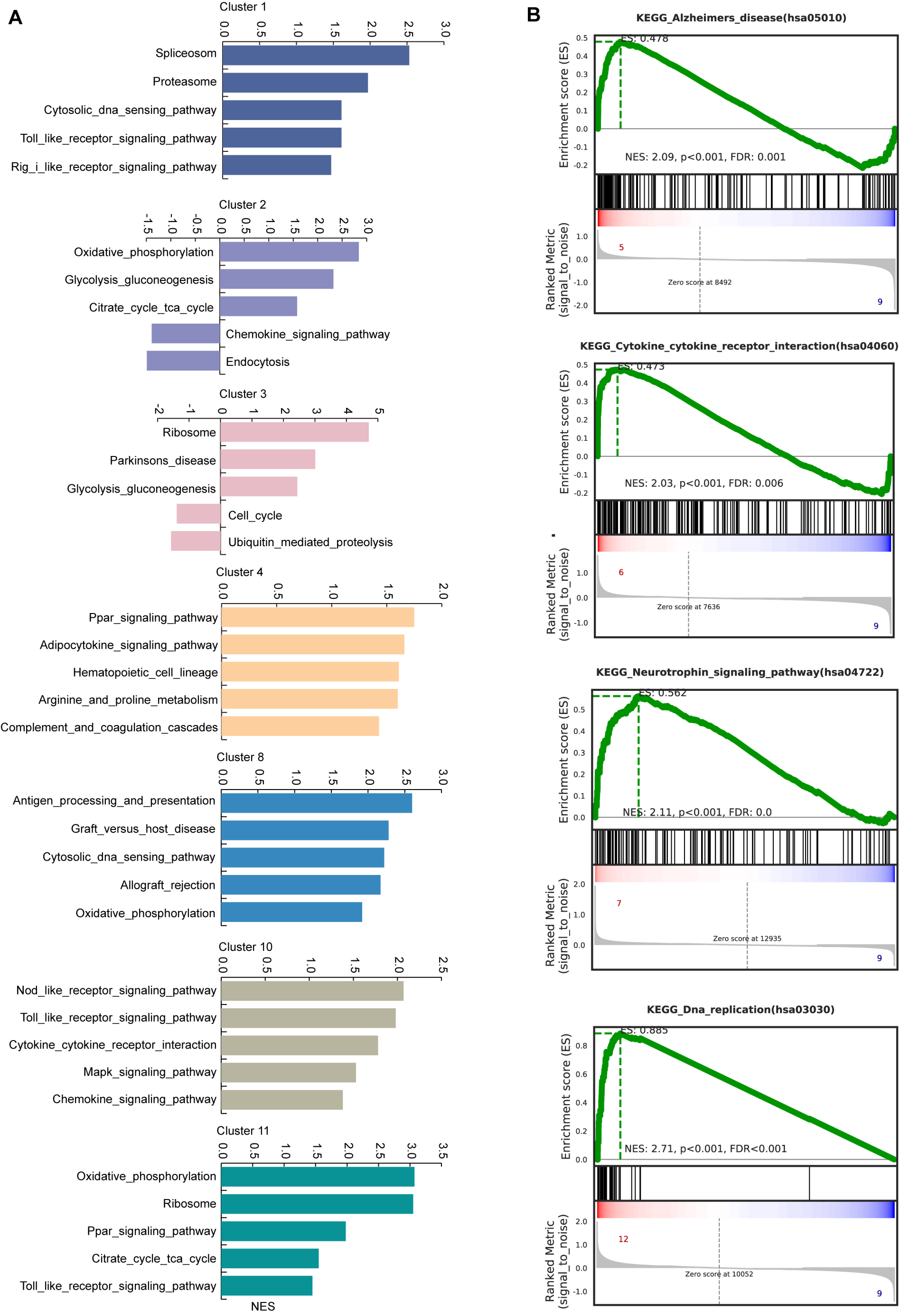

Supplement: Supplementary file 1 — Supplementary Material 1 [file 12974_2024_3113_MOESM1_ESM.png]

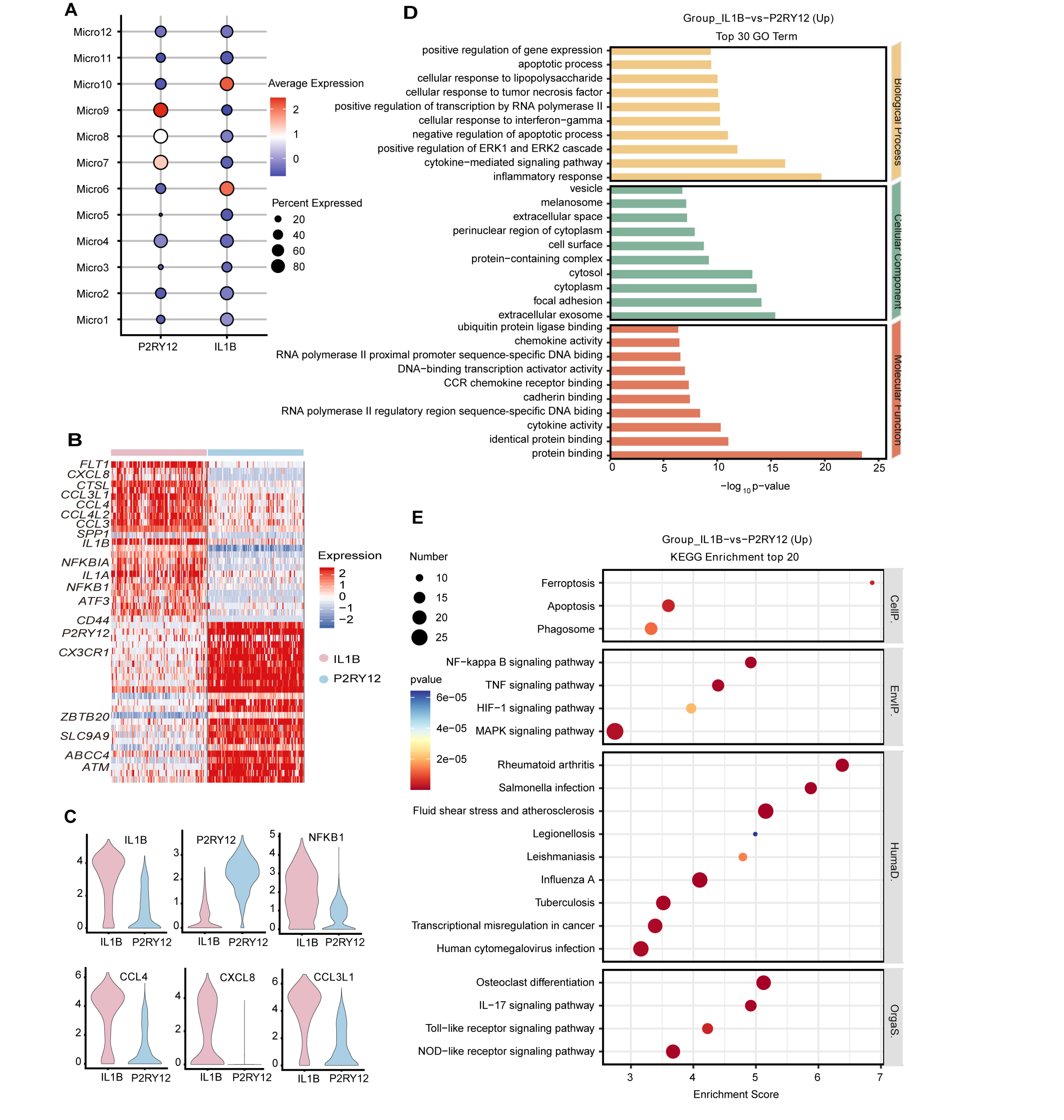

Supplement: Supplementary file 2 — Supplementary Material 2 [file 12974_2024_3113_MOESM2_ESM.png]

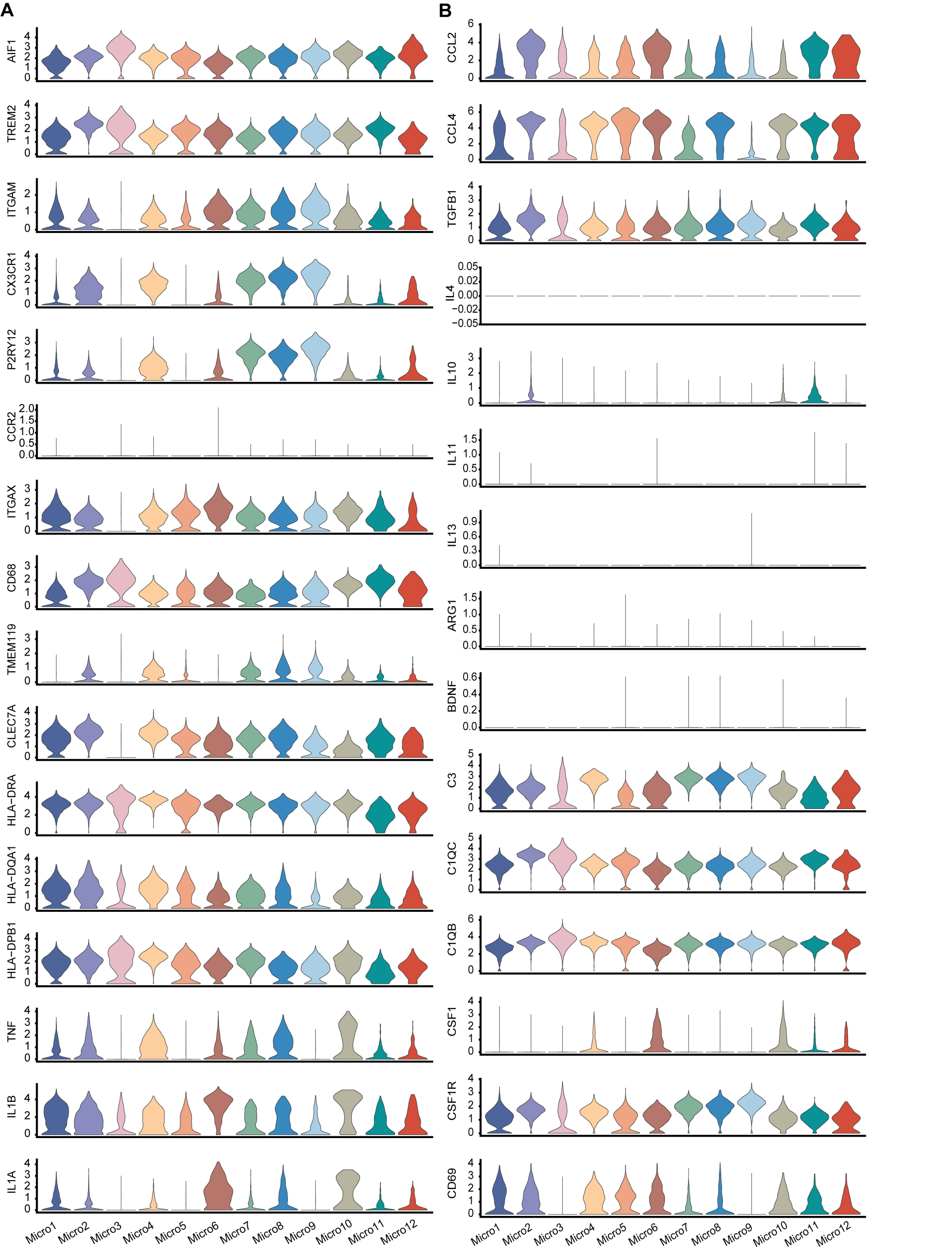

Supplement: Supplementary file 3 — Supplementary Material 3 [file 12974_2024_3113_MOESM3_ESM.png]

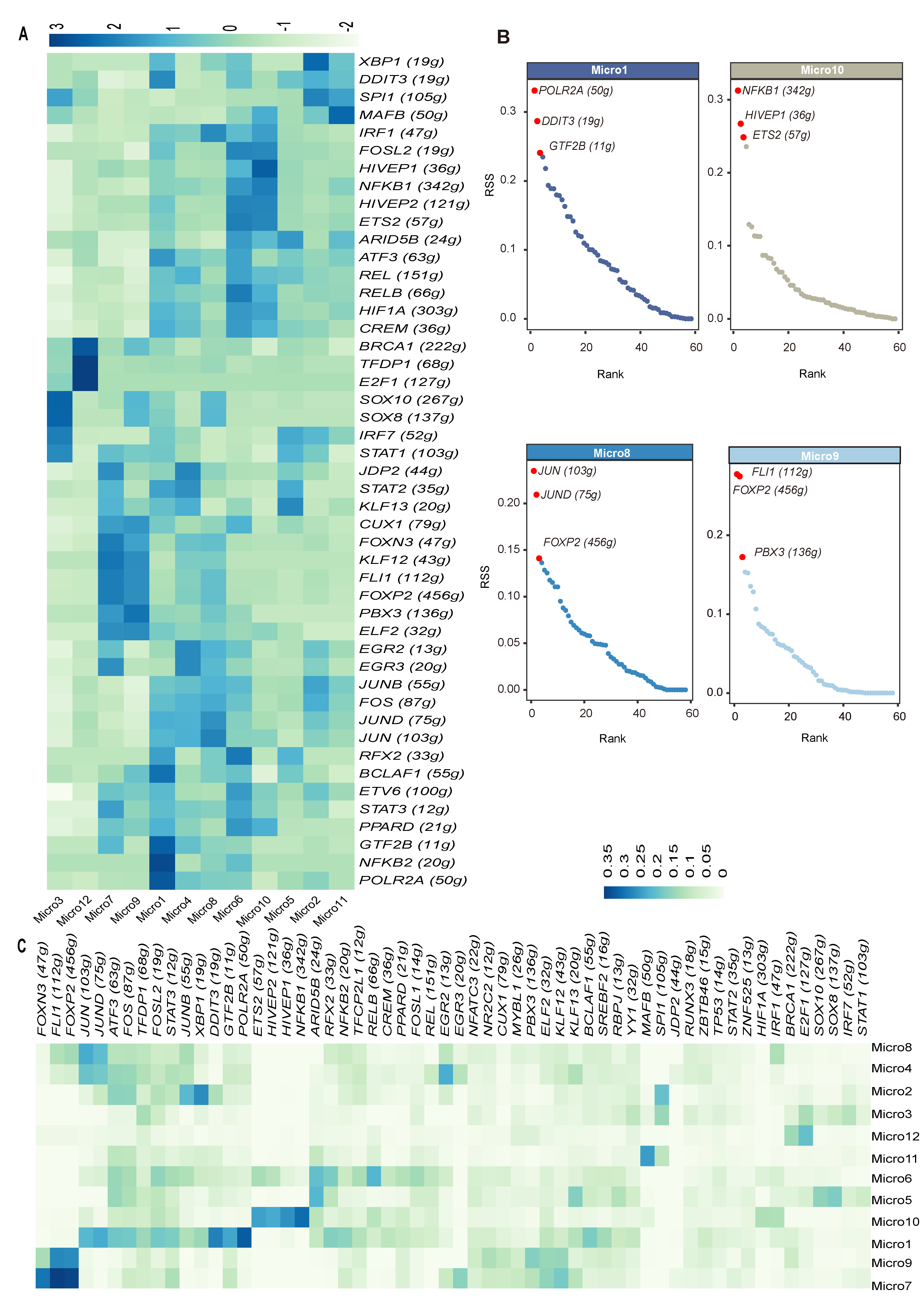

Supplement: Supplementary file 4 — Supplementary Material 4 [file 12974_2024_3113_MOESM4_ESM.png]

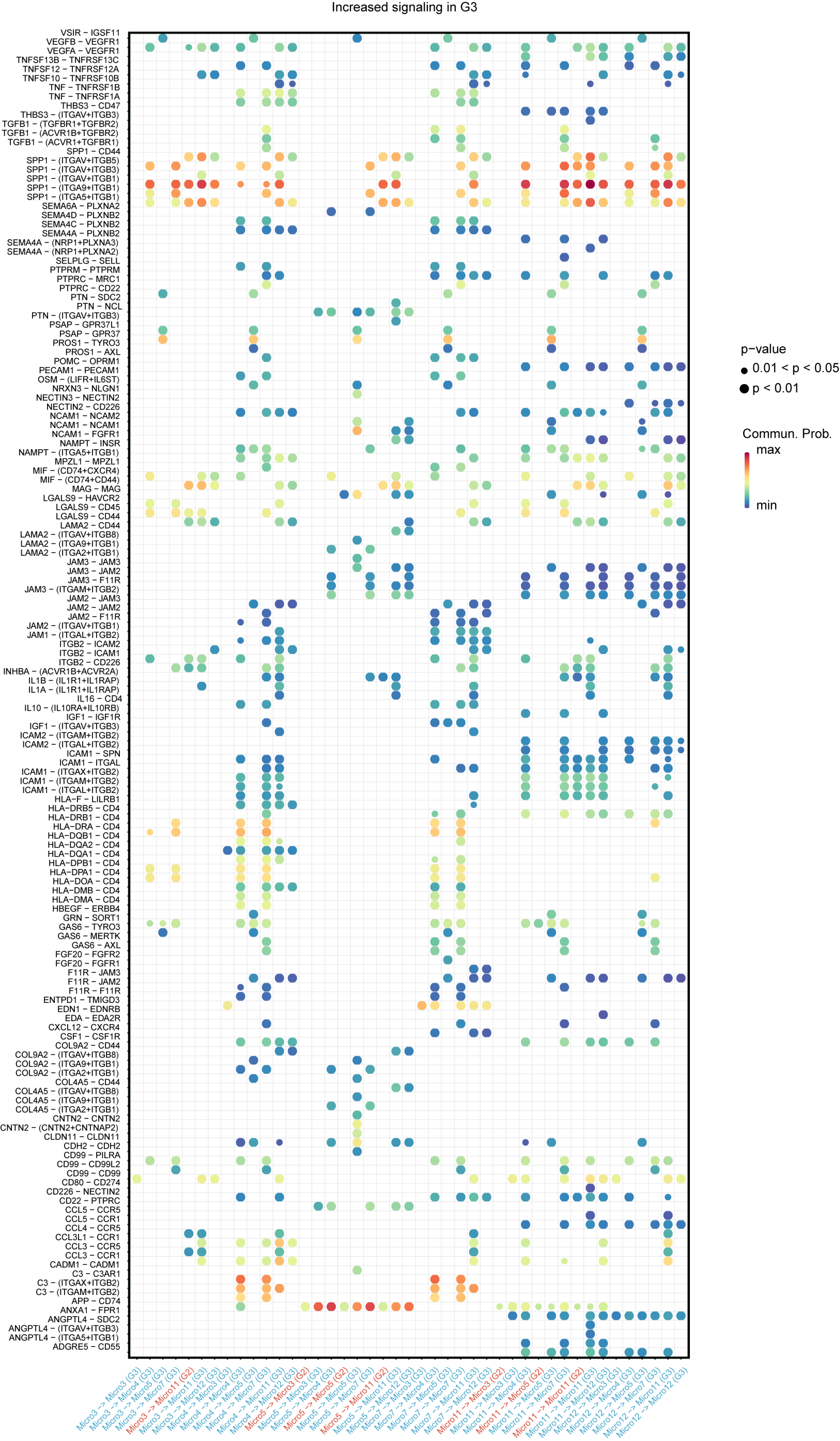

Supplement: Supplementary file 5 — Supplementary Material 5 [file 12974_2024_3113_MOESM5_ESM.png]

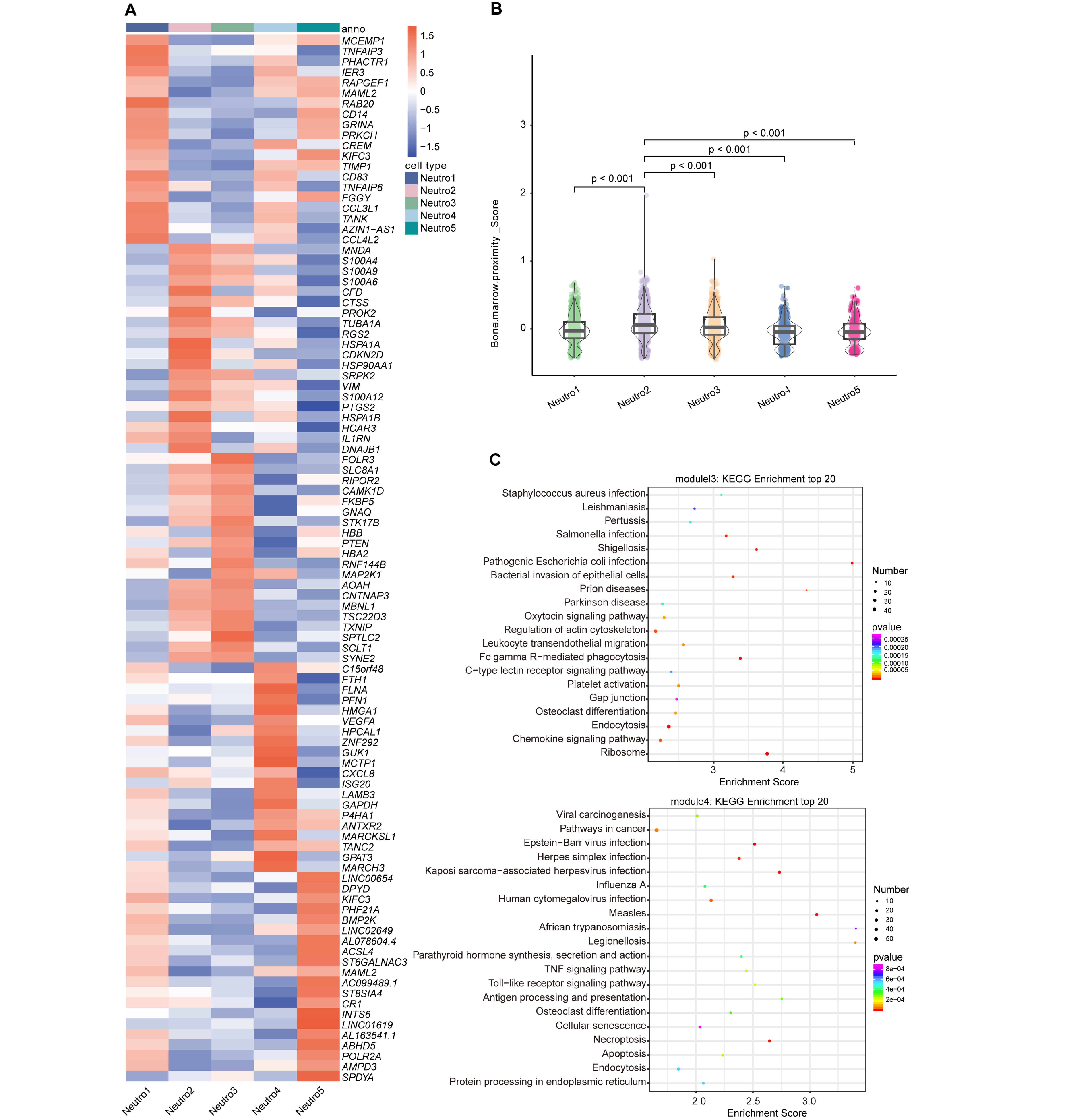

Supplement: Supplementary file 6 — Supplementary Material 6 [file 12974_2024_3113_MOESM6_ESM.png]

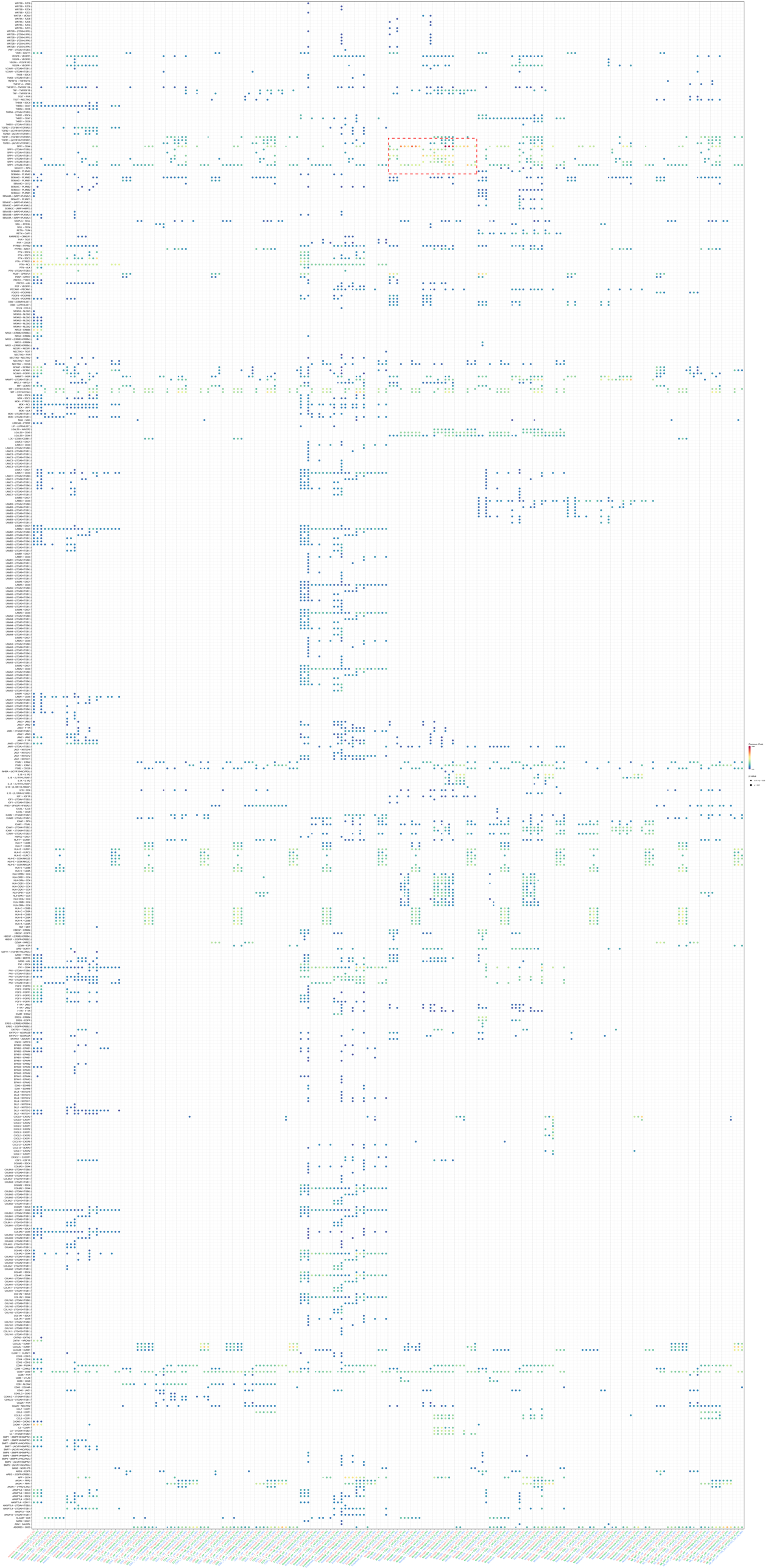

Supplement: Supplementary file 7 — Supplementary Material 7 [file 12974_2024_3113_MOESM7_ESM.pdf]
