# Supplementary material for: Single-cell RNA sequencing reveals the evolution of the immune landscape during perihematomal edema progression after intracerebral hemorrhage
Source: J Neuroinflammation. 2024 May 28;21:140. doi: 10.1186/s12974-024-03113-8 (PMC11131315; doi:10.1186/s12974-024-03113-8)
Supplement: Supplementary file 8 — Supplementary Material 8 [file 12974_2024_3113_MOESM8_ESM.docx]

| Characteristics | G1 (n=3) | G2 (n=3) | G3 (n=3) |
| --- | --- | --- | --- |
| Age （mean±SD） | 61±5.57 | 63±5.51 | 62±12.6 |
| Male | 33.33% | 66.66% | 66.66% |
| Hypertension | 100% | 100% | 100% |
| Stroke | 0 | 0 | 0 |
| Diabetes | 0 | 33.33% | 0 |
| GCS score | 7.67±1.52 | 6.67±2.08 | 6.00±1.73 |
| Systolic Blood Pressure | 188±11.8 | 185±20.0 | 175±25.0 |
| Hematoma Volume | 53±4.36 | 55±4.58 | 55±4.04 |

Table 1 Baseline Values
