# Supplementary material for: Single-cell RNA sequencing reveals the evolution of the immune landscape during perihematomal edema progression after intracerebral hemorrhage
Source: J Neuroinflammation. 2024 May 28;21:140. doi: 10.1186/s12974-024-03113-8 (PMC11131315; doi:10.1186/s12974-024-03113-8)
Supplement: Supplementary file 13 — Supplementary Material 13 [file 12974_2024_3113_MOESM13_ESM.docx]

Fig. S1. Microglia states have diverse biological pathway correlates. **(A)** GSEA analysis of genes that differentiate each cluster from cluster 9 ('homeostatic microglia') suggests distinct biological pathways. **(B)** GSEA shows top enriched pathways in some microglial subclusters.

Fig. S2. IL-1B expressing microglia genes expression profile compared with P2RY12 expressing microglia. **(A)** Dotplot shows the normalized expression of P2RY12 and IL1B in microglia clusters of PHE tissue in patients with ICH. Dot size reflects the percentage of cells that showed genes, while color shows the expression levels of genes. **(B)** Heatmap of genes significantly modulated in IL-1B expressing clusters compared to P2RY12 expressing clusters. For the heatmap, 100 randomly sampled cells were shown for both IL1B and P2RY12 clusters. **(C)** P2RY12, IL1B clusters marker, chemokine CCL4, CCL3L1, and proinflammatory nuclear transcription factor *NFKB1* were shown as violin plots. **(D)** GO gene set enrichment analysis results were shown as a bar plot where the x-axis -log of FDR adjusted p-value for GO terms was shown. The top 30 GO terms are shown in this figure. **(E)** KEGG gene set enrichment analysis results were shown as bubble plots. The top 20 KEGG terms are shown in the figure.

Fig. S3. Characterizing the gene expression profile of selected microglia-specific, inflammatory, and activation marker genes. **(A-B)** The x-axis shows microglia subclusters, and the y-axis shows normalized expression levels.

Fig. S4. Transcription factor regulatory networks are specific to microglia phenotypes. **(A)** SCENIC workflow identified transcription factor-regulated networks associated with different phenotypic clusters of microglia. Heatmap of each microglia subtype's inferred regulon activity score (RAS) in cluster levels. **(B)** Ranking plot of regulon specificity score (RSS). The higher RSS of the regulon may be specific to the subtypes. **(C)** Heatmap of regulon specificity score (RSS). The higher RSS of the regulon may be specific to the subtypes.

Fig. S5. Signaling changes of microglia subcluster during PHE tissue progression. Cell ligand-receptor inference analysis of microglial subtypes during PHE tissue progression (G3 vs. G2).

Fig. S6. The heterogeneity of neutrophils during PHE progression**.** **(A)** Differential expression analysis comparing each cluster to all others demonstrates distinct gene expression profiles. The top 20 genes from each cluster are displayed with gene names annotated on the right. **(B)** Violin plot showing the scores of functional modules for each neutrophil subcluster, using the AddModuleScore function. **(C)** Enriched KEGG terms for gene sets from four modules (modules 3 and 4) were represented on the left side.

Fig. S7. Cell ligand-receptor inference analysis of immune cells during PHE progression. Bubble plot of the significant differentially expressed ligand–receptor pairs during PHE progression. Dot color reflects communication probabilities, and dot size represents computed p-values. Empty space means the communication probability is zero. The p-values were computed from a two-sided permutation test.
